# Developmental transitions in amygdala PKC isoforms and AMPA receptor expression associated with threat memory in infant rats

**DOI:** 10.1038/s41598-018-32762-y

**Published:** 2018-10-02

**Authors:** Maya Opendak, Roseanna M. Zanca, Eben Anane, Peter A. Serrano, Regina M. Sullivan

**Affiliations:** 1 Emotional Brain Institute, Nathan Kline Institute, Orangeburg, 10962 USA; 2 Child Study Center, Child & Adolescent Psychiatry, New York University School of Medicine, New York, 10016 USA; 3 Department of Psychology, CUNY Hunter College, New York, 10016 USA; 4 The Graduate Center of CUNY, New York, 10016 USA

## Abstract

Although infants learn and remember, they rapidly forget, a phenomenon known as infantile amnesia. While myriad mechanisms impact this rapid forgetting, the molecular events supporting memory maintenance have yet to be explored. To explore memory mechanisms across development, we used amygdala-dependent odor-shock conditioning and focused on mechanisms important in adult memory, the AMPA receptor subunits GluA1/2 and upstream protein kinases important for trafficking AMPAR, protein kinase M zeta (PKMζ) and iota/lambda (PKCι/λ). We use odor-shock conditioning in infant rats because it is late-developing (postnatal day, PN10) and can be modulated by corticosterone during a sensitive period in early life. Our results show that memory-related molecules did not change in pups too young to learn threat (PN8) but were activated in pups old enough to learn (PN12), with increased PKMζ-PKCι/λ and GluA2 similar to that observed in adult memory, but with an uncharacteristic decrease in GluA1. This molecular signature and behavioral avoidance of the conditioned odor was recapitulated in PN8 pups injected with CORT before conditioning to precociously induce learning. Blocking learning via CORT inhibition in older pups (PN12) blocked the expression of these molecules. PN16 pups showed a more adult-like molecular cascade of increased PKMζ-PKCι/λ and GluA1–2. Finally, at all ages, zeta inhibitory peptide (ZIP) infusions into the amygdala 24 hr after conditioning blocked memory. Together, these results identify unique features of memory processes across early development: AMPAR subunits GluA1/2 and PKC isoform expression are differentially used, which may contribute to mechanisms of early life forgetting.

## Introduction

**Figure 1:**
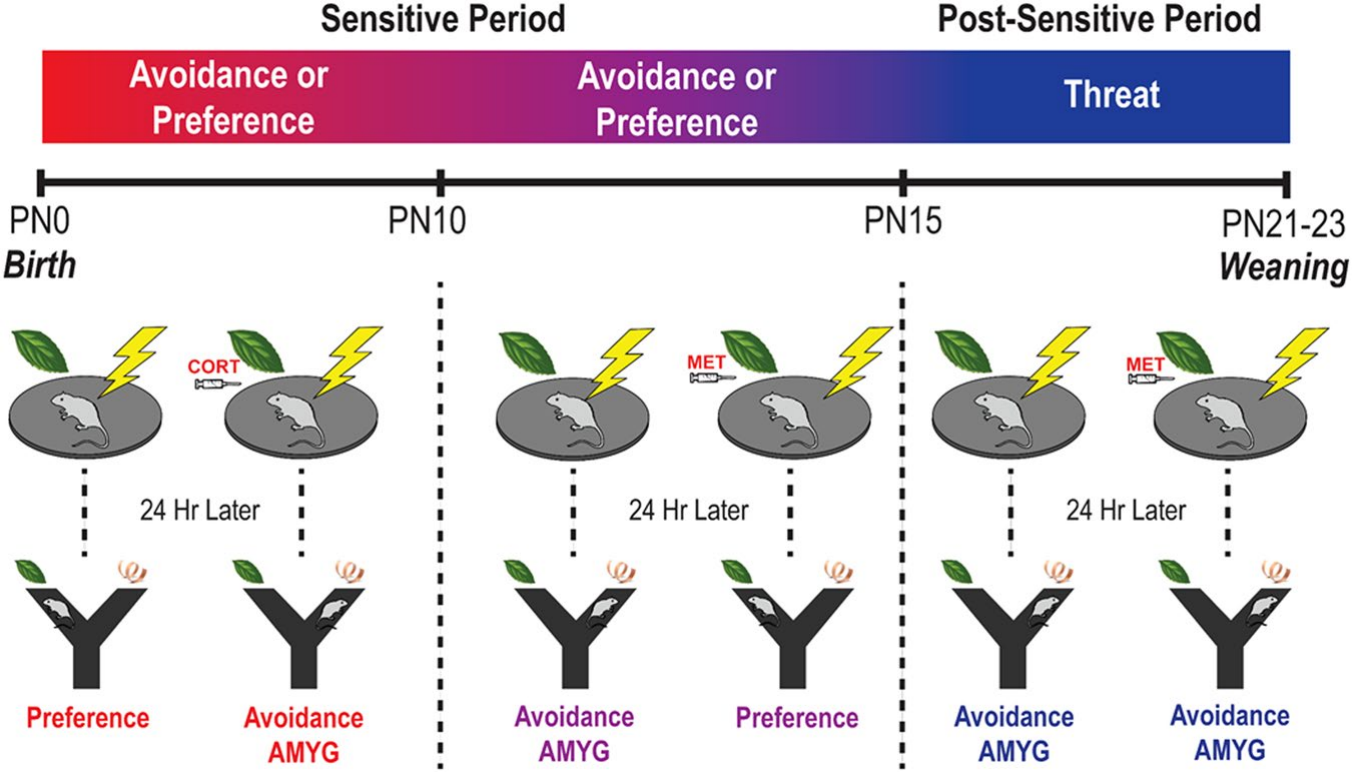
Schematic of developmental framework and experimental design. This set of studies capitalizes on known developmental milestones in infant amygdala-dependent threat learning to assess the underlying neural mechanisms of infant memory development. Pup amygdala-dependent threat learning shows developmental transitions and a unique dependence on the stress hormone corticosterone (CORT). At PN10, amygdala-dependent threat learning emerges in infant rats^18^. Before PN15, this learning can be blocked if CORT levels are inhibited^90,92^ and induced if CORT levels are increased. Specifically, in pups younger than PN16, amygdala CORT must be present to support avoidance learning: if CORT is low (e.g. via metyrapone (MET) injection), this avoidance learning will be blocked and amygdala-independent preference for the odor is observed. On the other hand, elevated amygdala CORT will permit amygdala-dependent threat learning, which occurs naturally in pups older than PN10 and can be induced in pups <PN10 via injection, a fearful mother or early life maltreatment^23^. Because CORT can turn learning on/off before PN15, we term this learning “Sensitive Period”. After PN15, “Post-Sensitive Period”, the ability of CORT to switch amygdala-dependent learning on/off has terminated^58^. At this time, conditioning with natural shock-induced CORT increases or with CORT blocker (MET) does not switch off avoidance learning and all pups learn and remember to avoid the odor. Here, we employ Pavlovian conditioning in rats in the sensitive period and post-sensitive periods and test memory in a Y-maze 24 hours after conditioning.

Infants and young children can learn and remember, although this information is typically only transiently retained^1–7^. Evidence suggests this is not due to poor encoding of information, since younger children given equivalent learning or overlearning still forget^8,9^. This rapid forgetting, sometimes referred to as infantile amnesia, has been shown to be a robust phenomenon that is present in myriad learning systems and myriad species^7,10–14^. Much of the neurobehavioral research has focused on mechanisms using rodents, including the now classic demonstrations of infantile amnesia. These studies used conditioned fear in infant to adult rats to show that retention lasted days in the youngest animals, which slowly increases as pups mature^15–17^. Here, we approach assessment of early life memory and forgetting by exploring the development of the molecular mechanisms maintaining memory using rat pups from postnatal days (PN) 8–16. We expand upon the original research presented in seminal work by the Campbell and Spear laboratories in the 1970s and by using the developing aversion learning system in infant rats^15–17^. Specifically, we use classically conditioned amygdala-dependent odor-shock aversion, which functionally emerges at PN10^18^ and has the advantage of being precociously engaged in younger pups by increasing corticosterone (CORT) levels or switched off in older pups by decreasing CORT^19^ during a sensitive period in development (younger than PN16, see Fig. 1 for summary). During this period, pups’ CORT levels are typically controlled by the mother: a calm mother decreases pups’ CORT levels, a process termed social buffering^19–22^, while a fearful mother increases the pups’ CORT^23^. Thus, as we assess the molecular events supporting maintenance of memory during testing 24 hr after conditioning, we can control and disassociate the confounding shifts in the molecular cascade due to maturation: memory molecules were assessed in the same age animals that were odor-shock conditioned the previous day but learning switched on/off, by manipulating CORT.

The neurobiology of memory focuses on events that occur as long-term memory processes emerge and memories stabilize. Using adults, this memory stabilization process is thought to involve the rearrangement of synapses, partly mediated by α-amino-3-hydroxy-5-methyl-4-isoxazolepropionic acid (AMPA) receptors (AMPAR). Indeed, trafficking-mediated rapid intracellular translocation, receptor degradation or its prevention, and synapse stabilization all appear important^24–28^. These processes appear partly mediated by GluA1 and GluA2- containing AMPAR. In summary, AMPAR trafficking is well-documented to be a primary cellular mechanism employed in synaptic plasticity supporting memory,^29^ with irregular AMPAR regulation associated with memory impairments^30^.

In a process not yet completely understood, these AMPAR changes are initiated, at least in part, by kinase involvement in memory maintenance^31–36^. Although Ca2+/calmodulin-dependent protein kinase II (CaMKII) has also been identified as important in memory maintenance in adults^37–39^, we focused on PKMζ-PKCι/λ because infant levels are high and remain relatively unchanged during development, while CaMKII levels are low^40,41^. Specifically, PKMζ appears to facilitate memory maintenance by receptor trafficking to the synapse and increasing the number of AMPAR^42–47^. PKMζ has been implicated in the maintenance of amygdala-dependent memories of threat, with its post-learning inhibition eliminating memory^34,48–53^. Recent research suggests that under conditions of low PKMζ, PKCι/λ can serve as a compensatory mechanism^47,54,55^, and CaMKII research^37,56^ suggests there may be multiple pathways for memory within the brain. In the present work, we focus on synaptic PKMζ-PKCι/λ expression to begin our assessment of early life molecular mechanisms of memory.

Here we show a novel mechanistic description of memory maintenance in the developing infant rodent. Our results suggest that behavioral emergence of amygdala-dependent threat memory after PN10 coincides with an emerging causal role for AMPAR trafficking and their recruitment by PKMζ-PKCι/λ into memory maintenance, suggesting recruitment of adult-like memory mechanisms. However, the pathway to the adult mechanisms shows at least two transitions, including the transition from PKCι/λ to the dual activation of PKMζ-PKCι/λ and a switch from decreased to increased GluA1 supporting memory maintenance. The partial recruitment of adult molecular mechanisms in the infant may represent one mechanism for increased forgetting in early life.

## Results

### Experiment 1

#### Developmental emergence of intracellular AMPAR trafficking within the amygdala is associated with the developmental emergence of memory

**Figure 2:**
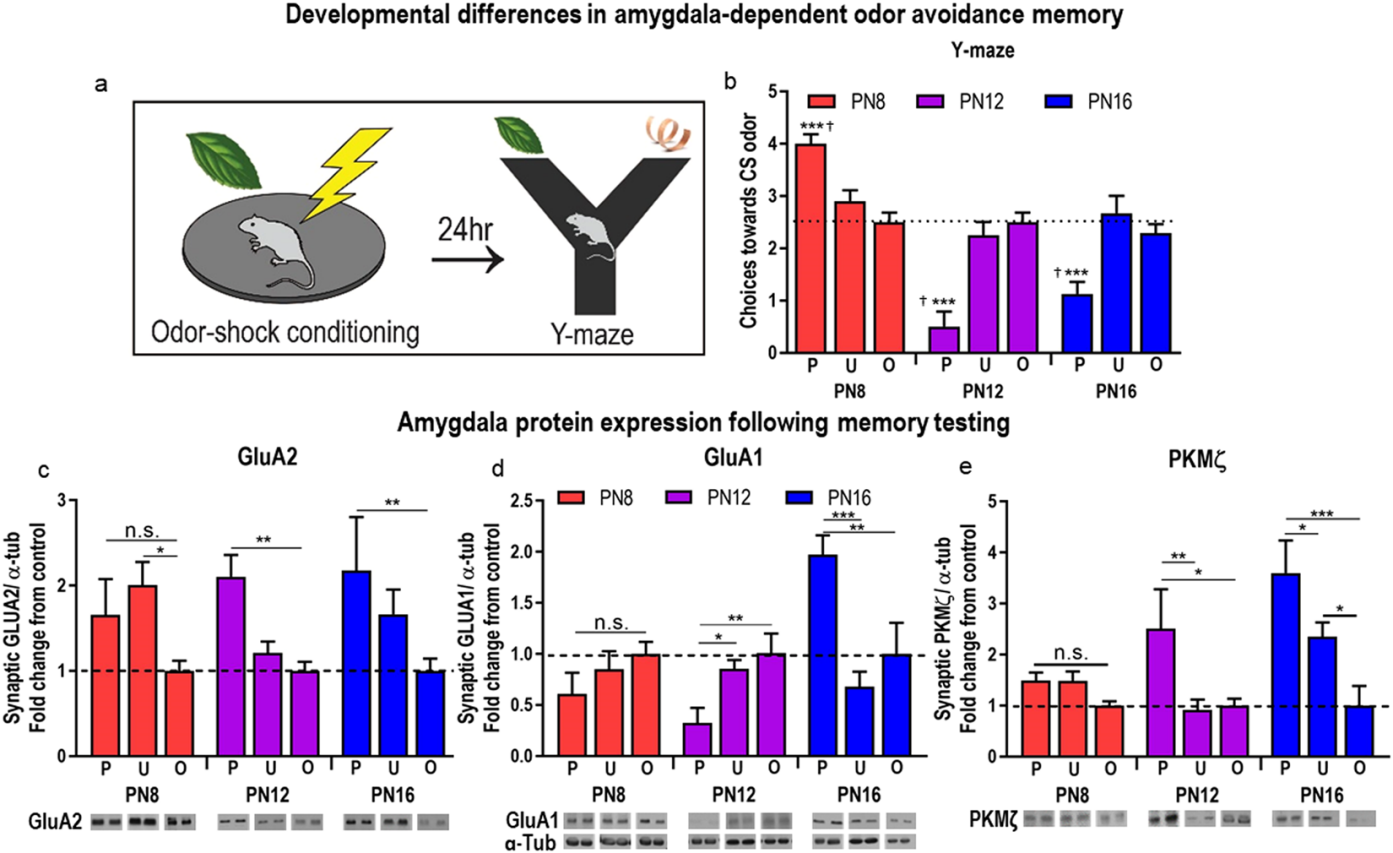
Developmental emergence of amygdala-dependent threat memory and amygdala protein expression. 24 hours following a Pavlovian odor-shock conditioning procedure that paired neutral peppermint odor presentations with 0.5 mA shocks to the tail, rat pups were tested for long-term memory using a Y-maze odor choice test containing the conditioned stimulus odor (peppermint) and a familiar odor (clean wood shavings) (**a**). Threat learning and memory emerged by PN12, as indicated by a significant reduction in choices towards the conditioned stimulus (CS) peppermint odor in the Y-maze choice test (**b**). Threat memory and corresponding avoidance behavior were associated with increases in PKMζ and GluA2 expression in the amygdala at PN12 and PN16 (**c**–**e**). GluA1 decreased in PN12 pups while it increased in PN16 pups (**d**). P/U/O: paired, unpaired, odor only. *P < 0.05, **p < 0.01, ***p < 0.001; ^†^ denotes significant difference from chance performance on Y-maze (dotted line), p < 0.05. Error bars indicate mean ± SEM. Dashed line: average protein expression in untrained “odor only” group (control). Blots for each condition show two adjacent lanes. The same tubulin-corrected values were used for all markers probed. Comparisons were made across gels processed in parallel using samples derived from the same experiment. Full-length blots/gels are presented in Supplementary Figures 1–5.

Behavioral emergence of memory: Consolidated memory of amygdala-dependent aversive odor-shock conditioning first emerges in rat pups at PN10^18^, as evidenced by the PN12 pup’s avoidance behavior to the conditioned odor stimulus (CS) 24 hr after training (Fig. 2a,b). Pups’ CS choices in the Y-maze changed as a function of age and condition [two-way ANOVA, interaction between age and condition, *F*_(4,55)_ = 18.15, *p* < 0.001, main effect of age (*F*_(2,55)_ = 29.13, *p < *0.001), main effect of condition (*F*_(2,55)_ = 7.931, *p* = 0.0009)]. *Post hoc* tests indicated that pups avoided the CS odor significantly more than the familiar odor (clean wood shavings used for bedding) at postnatal day (PN) 12 and PN16 relative to PN8 pups (PN8 vs. PN12, *p < *0.0001; PN8 vs. PN16, *p* < 0.0001; PN12 vs. PN16, *p* = 0.2089). Furthermore, PN8 pups that underwent paired odor-shock conditioning displayed a long-term preference for the CS odor (*t*_(7)_ = 7.937, *p* < 0.0001), while PN12 and PN16 displayed an aversion to the CS odor after paired conditioning (PN12, *t*_(6)_ = 5.203, *p* = 0.002; PN16, *t*_(11)_ = 6.435, *p* < 0.0001), as indicated by choices toward the odor at levels significantly greater or lesser than chance, respectively. This behavioral data replicates previous research^18,57,58^.

GluA2 is increased in PN12 and PN16 pups that remember threat: At both PN12 and PN16, GluA2 is increased 24 hr after learning in paired odor-shock groups compared to groups only receiving odor presentations [Fig. 2c; two-way ANOVA (age × condition [paired, unpaired, odor only]), main effect of condition: *F*_(2,43)_ = 9.882, *p* = 0.0066]. *Post hoc* tests revealed significant differences in GluA2 with memory at PN12 (paired vs. unpaired, *p* = 0.0099, paired vs. odor only, *p* = 0.0013) and PN16 (paired vs. odor only, *p* = 0.0054). No significant differences were observed with paired odor-shock conditioning at PN8 (paired vs. unpaired, *p* = 0.3744, paired vs. odor only, *p* = 0.1431), though we observed an increase in the unpaired condition vs. odor only (*p* = 0.0134). There were no differences between paired conditions at PN12 vs. PN16 (*p* = 0.8570).

GluA1 is decreased in PN12 pups that remember threat, decreased in PN16 pups that remember threat and unchanged in PN8 pups that do not remember threat: We observed an age-dependent change in postsynaptic GluA1 in animals that remembered to avoid peppermint 24 hr after conditioning [Fig. 2d; two-way ANOVA (age × condition [paired, unpaired, odor only]), significant interaction between age and condition: *F*_(4,48)_ = 8.836, *p* < 0.0001 and main effect of age (*F*_(2,48)_ = 5.748, *p* = 0.0058)]. *Post hoc* tests revealed a decrease in synaptic GluA1 in pups that had received paired odor-shock presentations at PN12 compared to odor only (paired vs. unpaired, *p* = 0.0110; paired vs. odor only, *p* = 0.0229). In contrast, pups that were conditioned at PN16 showed an *increase* in GluA1 24 hr later (paired vs. unpaired, *p* < 0.0001; paired vs. odor only, *p* = 0.0001). GluA1 expression did not differ 24 hr after conditioning in PN8 pups (paired vs. unpaired, *p* = 0.4892, paired vs. odor only, *p* = 0.3166).

PKMζ is increased in PN12 and PN16 pups that remember threat and did not change in PN8 pups that do not remember threat: Amygdala-dependent threat memory of the odor cue 24 hr after conditioning at PN12 and PN16 was associated with increased postsynaptic PKMζ (Fig. 2e; two-way ANOVA [age × condition (paired, unpaired, odor only)], main effects of age: *F*_(2,42)_ = 4.886, *p* = 0.0124; main effect of condition: *F*_(2,42)_ = 9.665, *p* = 0.0004). *Post hoc* tests revealed significant differences in PKMζ associated with threat memory at PN12 (paired vs. unpaired, *p* = 0.0057, paired vs. odor only, *p* = 0.0108) and PN16 (paired vs. unpaired, *p* = 0.0415, paired vs. odor only, *p* < 0.0001, unpaired vs. odor only, *p* = 0.0273). No significant differences were observed between memory conditions at PN8 (paired vs. unpaired, *p* = 0.9865, paired vs. odor only, *p* = 0.4807) although a trend was observed between paired conditions at PN12 and PN16 (*p* = 0.0594). PN8 also showed no significant changes in cytosolic PKMζ (data not shown).

In sum, using amygdala-dependent odor-shock conditioning, we found that mechanisms important in adult memory did not change in pups too young to show this memory (PN8). In pups old enough to remember threat, adult-like mechanisms associated with postsynaptic PKMζ and GluA2 increased, although GluA1 showed age- related changes in learning, with a decrease at PN12 and an increase at PN16. In the next experiment, we dissociated maturation and learning by switching on precocious learning at PN8 via CORT increase, switching off learning at PN12 via a CORT decrease, and manipulating CORT without switching on/off learning at PN16.

### Experiment 2

#### Dissociating maturation and learning by switching learning on/off with CORT: Continued alignment with memory-related AMPAR expression

A major confound of developmental research is that the landscape of the molecular cascade changes. Here we dissociate the confounding shifts in the molecular cascade supporting memory due to maturation versus learning: memory molecules were assessed in the same age animals that were odor-shock conditioned the previous day but learning switched on/off, by manipulating CORT. The pharmacological effects of our CORT injection or its blockade manipulations are well-documented to last only a few hours^59,60^.

**Figure 3:**
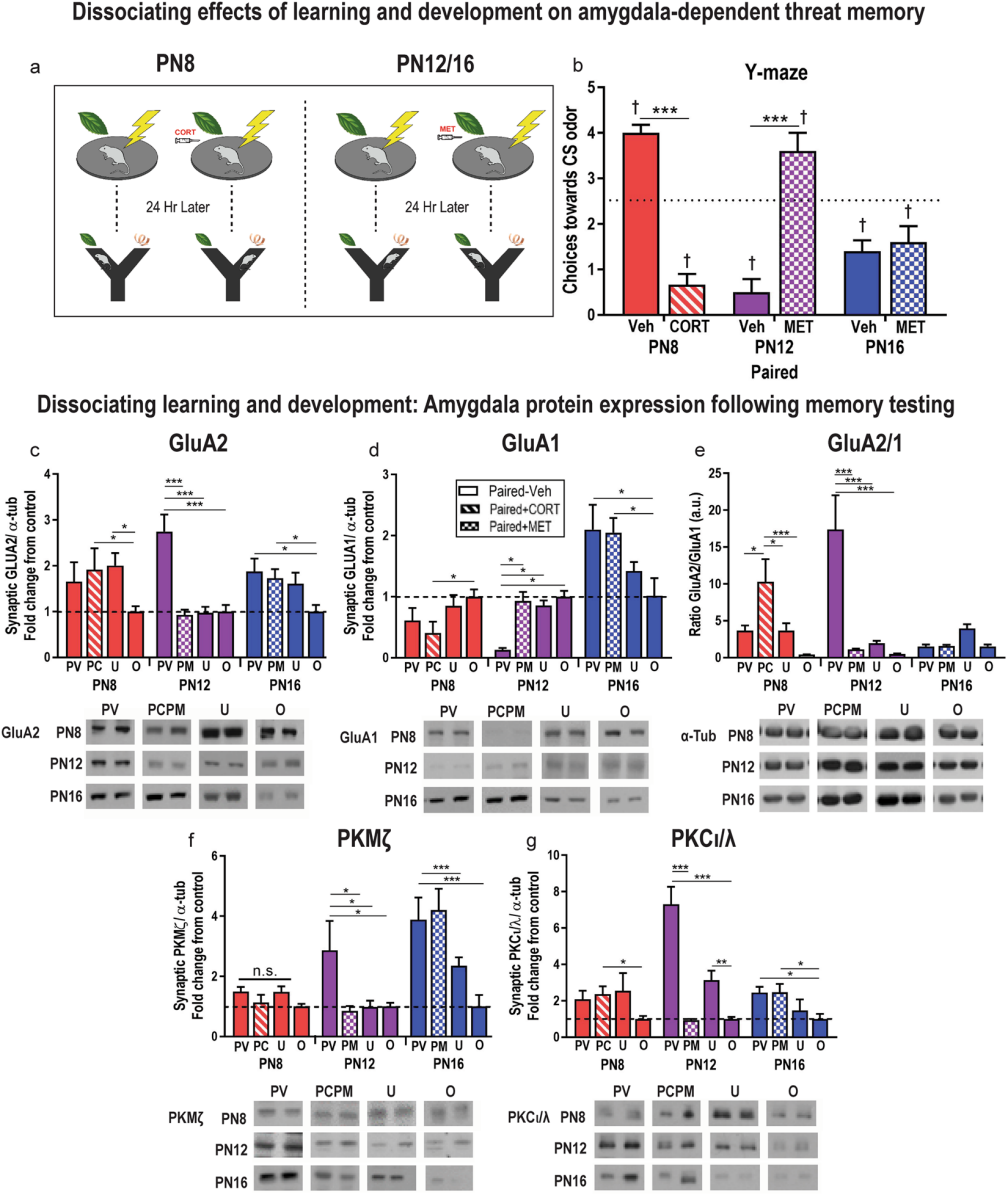
Dissociating effects of learning and development on amygdala-dependent threat memory. PN12 and PN16 pups were injected i.p. with CORT blocker (metyrapone, MET) or vehicle solution and PN8 pups were injected with CORT or vehicle before training and tested for long-term memory retention in the Y-maze 24 hr later (**a**). MET prevented long-term threat memory consolidation as assessed in the Y-maze at PN12, but not PN16, while CORT administration produced an avoidance in PN8 pups (**b**). MET delivered i.p. prevented learning at PN12, but not PN16, and prevented GluA1/2 changes observed in PN12 vehicle-injected controls. CORT administration in PN8 pups before conditioning produced avoidance of the CS 24 hr later and changes in GluA1/2 observed in PN12 pups given vehicle injections (**c**,**d**). GluA2/1 ratio was increased 24 hr after conditioning in PN8 pups that received CORT and PN12 pups that received vehicle injections (**e**). MET injections at PN12 prevented threat learning and prevented memory-associated increases in PKMζ, but memory-associated increases were preserved in PN16 pups conditioned with MET and vehicle. Threat-induced learning via CORT in PN8 pups failed to increase PKMζ (**f**). PKCι/λ expression was increased in pups that showed avoidance memory in the Y maze (PN8 PC, PN12 PV, PN16 PV/PM) (**g**). *P < 0.05, **p < 0.01, ***p < 0.001; ^†^ denotes significant difference from chance performance on Y-maze (dotted line), p < 0.05. Error bars indicate mean ± SEM. PV/PM/PC/U/O: paired + vehicle, paired + metyrapone, paired + CORT, unpaired, odor only. Dashed line: average protein expression in untrained “odor only” group (control). Blots for each condition show two adjacent lanes. The same tubulin-corrected values were used for all markers probed. Comparisons were made across gels processed in parallel using samples derived from the same experiment. Full-length blots/gels are presented in Supplementary Figures 1–5.

It has been shown that during a sensitive period in development (<PN16), plasma CORT levels can control whether infants learn to avoid an odor paired with shock^19,57^, with CORT blockade preventing avoidance/threat learning from PN10–15 and CORT administration producing precocious threat learning in pups < PN10 (summarized in Fig. 1). Here, we explored the effects of CORT modulation of learning on mechanisms of memory maintenance in the amygdala. PN12 and PN16 infants were given systemic injections of CORT blocker (metyrapone [MET], 50 mg/kg; Sigma-Aldrich, St. Louis, MO) or vehicle solution 90 minutes before conditioning, and tested for long-term memory retention in the Y-maze 24 hr later. In addition, PN8 pups were either administered CORT (3 mg/kg, Sigma-Aldrich, St. Louis, MO) or vehicle 30 minutes before paired odor-shock conditioning, tested in the Y-maze 24 hr later and brains harvested (Fig. 3a,b).

Decreasing CORT via MET injection blocked learning in PN12 pups, but had no effect on PN16 pups. Increasing CORT in PN8 odor-shock pups produced an odor aversion memory: During long-term memory testing, MET-injected pups approached the CS odor in the Y-maze significantly more than vehicle-injected control pups at PN12, but MET had no effect on choices at PN16 [Fig. 3b; two-way ANOVA (age × drug), significant interaction, *F*_(2,32)_ = 59.37, *p* < 0.0001, main effect of age, *F*_(2,32)_ = 4.109, *p* = 0.0258]. *Post hoc* tests showed that CORT inhibition during acquisition influenced Y-maze performance at PN12, but not PN16 (PN12 vehicle vs. drug, *p* = 0.001, PN16 vehicle vs. drug, *p* = 0.99). In fact, pups injected with CORT blocker (“MET” in graph) displayed a long-term preference for the CS odor (*t*_(7)_ = 3.06, *p* = 0.0185), while vehicle-injected pups displayed a long-term aversion to the CS odor, choosing it significantly below chance (*t*_(6)_ = 10.25, *p* < 0.0001, Fig. 3b). PN8 pups that received CORT (“CORT” in graph) showed avoidance of the odor stimulus in the Y-maze test, choosing it significantly below chance (*t*_(8)_ = 7.778, *p* < 0.0001) while PN8 pups receiving vehicle recapitulated the preference for the odor shown in pups receiving paired presentations without CORT administration (*t*_(7)_ = 7.937, *p* < 0.0001). Together, these results replicate behavioral results and engagement of the amygdala in learning^18,58^ and support the use of this conditioning system for assessment of memory.

Modulating learning via CORT maintained a strong alignment with the molecular cascade associated with memory: CORT-induced modulation of learning in PN8 and PN12 pups resulted in memory-associated changes in PKMζ/AMPAR expression 24 hr later. Specifically, CORT inhibition via metyrapone (MET) administration during acquisition (“PM” in graph, Fig. 3c–e) in PN12 pups prevented increases in PKMζ and GluA2 and prevented the decrease in GluA1 associated with memory at this age. PN16 pups that received MET before paired odor-shock conditioning maintained learning and associated increases in PKMζ, GluA2 and GluA1 (Fig. 3c). In contrast, administering CORT to PN8 pups before acquisition (“PC” in graph) produced learning and an increase in GluA2, a decrease in GluA1 and no change in synaptic or cytosolic (data not shown) PKMζ 24 hr later.

GluA2 changes remain associated with memory: Two-way ANOVA revealed a significant interaction between age and drug (*F*_(6,56)_ = 2.853, *p* = 0.0170) and a main effect of age (*F*_(3,56)_ = 7.213, *p* = 0.0004). *Post hoc* tests revealed at PN12, CORT inhibition in pups receiving paired odor-shock presentations reduced GluA2 levels equivalent to controls that only received presentations of the CS odor (PN12: paired + MET vs. odor only, *p* = 0.8439, paired + vehicle vs. odor only, *p* = 0.0002). PN8 pups that received CORT with paired presentations of odor-shock showed an increase in GluA2 expression compared to pups that only received the odor (*p* = 0.0331). In contrast, metyrapone administration to PN16 pups failed to prevent increases in GluA2 24 hr after odor-shock pairings (PN16: paired + MET vs. odor only, *p* = 0.0160; paired + vehicle vs. odor only, *p* = 0.0105).

GluA1 changes remain associated with memory, decreasing at PN8 and PN12 in pups that remember, and increasing at PN16 in pups that remember: GluA1 response to learning was similar in PN12 and CORT induced precocious learning in PN8 (Fig. 3d). Specifically, CORT administration to induce threat learning in PN8 pups produced a memory-associated GluA1 decrease 24 hr later, compared to pups that only received odor presentations (*p *= 0.0395). Importantly, blocking PN12 learning by CORT inhibition (via metyrapone) during acquisition prevented memory-associated GluA1 decreases 24 hr later. Furthermore, at PN16, an age when CORT blockade failed to block learning, memory-associated GluA1 increases were observed 24 hr after conditioning. Specifically, two-way ANOVA revealed a significant interaction between age and condition on GluA1 (*F*_(4,41)_ = 4.744, *p* = 0.0031) and a main effect of age (*F*_(2,41)_= 16.02, *p* < 0.0001). *Post hoc* testing revealed that in PN12 pups, MET administration, which prevented threat learning, blocked the memory-associated GluA1 decrease observed following paired odor-shock conditioning 24 hr later (PN12: paired + MET vs. odor only, *p* = 0.8618, paired + vehicle vs. odor only, *p* = 0.0297). Conversely, in PN16 where MET failed to prevent threat learning, it also failed to block the memory-associated increase in GluA1 observed 24 hr after conditioning with vehicle (paired + MET vs. odor only, *p* = 0.0054, paired + vehicle vs. odor only, *p* = 0.0029).

Ratio of GluA2 to GluA1 increases in young pups that exhibit threat memory: The ratio of GluA2 to GluA1 showed age and learning-dependent changes associated with threat memory (Fig. 3e). Two-way ANOVA revealed a significant interaction between age and condition on the GluA2/GluA1 ratio (*F*_(6,52)_ = 7.721, *p* < 0.001) and a main effect of condition (*F*_(3,52)_ = 4.924, *p* = 0.0044). *Post hoc* testing revealed significant differences between groups. CORT administration before paired conditioning in PN8 pups was associated with increased GluA2/GluA1 24 hr later compared to paired conditioning with vehicle (paired + CORT vs. paired + vehicle, *p* = 0.0405), unpaired (paired + CORT vs. unpaired, *p* = 0.02) and odor only (paired + CORT vs. odor only, *p* = 0.0028). In PN12 pups, paired odor-shock presentations with vehicle onboard produced increases higher than odor only (*p* < 0.0001), unpaired (*p* < 0.0001) and metyrapone-treated pups (*p < *0.0001). In PN16 pups, GluA2/1 ratio did not differ between any conditions (paired + MET vs. paired + vehicle, *p* = 0.9922, paired + MET vs. unpaired, *p* = 0.416, paired + MET vs. odor only, *p* = 0.9816, paired + vehicle vs. unpaired, *p* = 0.4640, paired + vehicle vs. odor only, *p* = 0.9994, unpaired vs. odor only, *p* = 0.4407). This analysis highlights the differential expression between GluA2 and GluA1 subunits in PN8 and PN12 indicative of dysregulated GluA2/1 heteromer expression that is associated with impaired synaptic plasticity^29^.

PKC isoforms increase with memory: PKMζ is associated with memory in older pups: Manipulating learning with CORT showed age-dependent effects on memory-associated PKMζ expression 24 hr later. Two-way ANOVA [age × condition (paired + MET/paired + vehicle/unpaired/odor only)] showed a significant interaction between the two factors on PKMζ expression (*F*_(6,56)_ = 2.864, *p* = 0.0167) and main effects of age (*F*_(2,56)_ = 11.97, p < 0.0001) and condition (*F*_(3,56)_ = 5.762, *p* = 0.0017). *Post hoc* tests revealed significant differences between groups. At PN12, CORT inhibition (blocks learning) in pups receiving paired odor-shock presentations reduced PKMζ levels equivalent to controls (PN12: paired + MET vs. odor only, *p* = 0.8523, paired + vehicle vs. odor only, *p* = 0.0141). In contrast, MET administration to PN16 pups (learning retained) retained the memory-associated PKMζ increase following odor-shock pairings (PN16: paired + MET vs. odor only, *p* < 0.0001; paired + vehicle vs. odor only, *p* < 0.0001). In PN8 pups, CORT administration (precociously induced learning) did not alter memory-associated PKMζ expression (paired + CORT vs. odor only, *p* = 0.8774; paired + vehicle vs. odor only, *p* = 0.5692). Thus, the increase in PKMζ in memory diverges between pups that are PN8 versus PN12 and PN16.

PKC isoforms increase with memory: PKCι/λ associated with memory in all aged pups: Figure 3g shows amygdala-dependent threat memory was associated with increases in PKCι/λ expression. Two-way ANOVA (age × condition) revealed an interaction between the two factors (*F*_(6,56)_ = 3.406, *p* = 0.0062) and a main effect of condition (*F*_(3,56)_ = 12.12, *p* < 0.0001). *Post hoc* tests showed that at PN12, pups that received both paired and unpaired presentations of odor-shock showed increased compared to odor only (paired + vehicle vs. odor, *p* = 0.0004, unpaired vs. odor, *p* = 0.0340). MET administration before paired conditioning prevented this increase (paired + MET vs. paired + vehicle, *p* < 0.0001). At PN16, paired conditioning was associated with increased PKCι/λ vs. odor only (paired + vehicle vs. odor only, *p* = 0.0156), which was not suppressed by MET administration (paired + MET vs. paired + vehicle, *p* = 0.8292, paired + MET vs. odor only, *p* = 0.0407). In PN8 pups, CORT administration before paired odor-shock conditioning was associated with increased PKCι/λ expression (paired + CORT vs. odor only, *p* = 0.0242). Paired presentations without CORT and unpaired presentations were not associated with statistically significant increases in PKCι/λ compared to untrained controls (paired + vehicle vs. odor only, *p* = 0.089, unpaired vs. odor only, *p* = 0.208). Thus, the increases in PKCι/λ in memory remains constant across ages.

### Experiment 3

#### PKC isoforms are causal in amygdala-dependent threat memory maintenance

**Figure 4:**
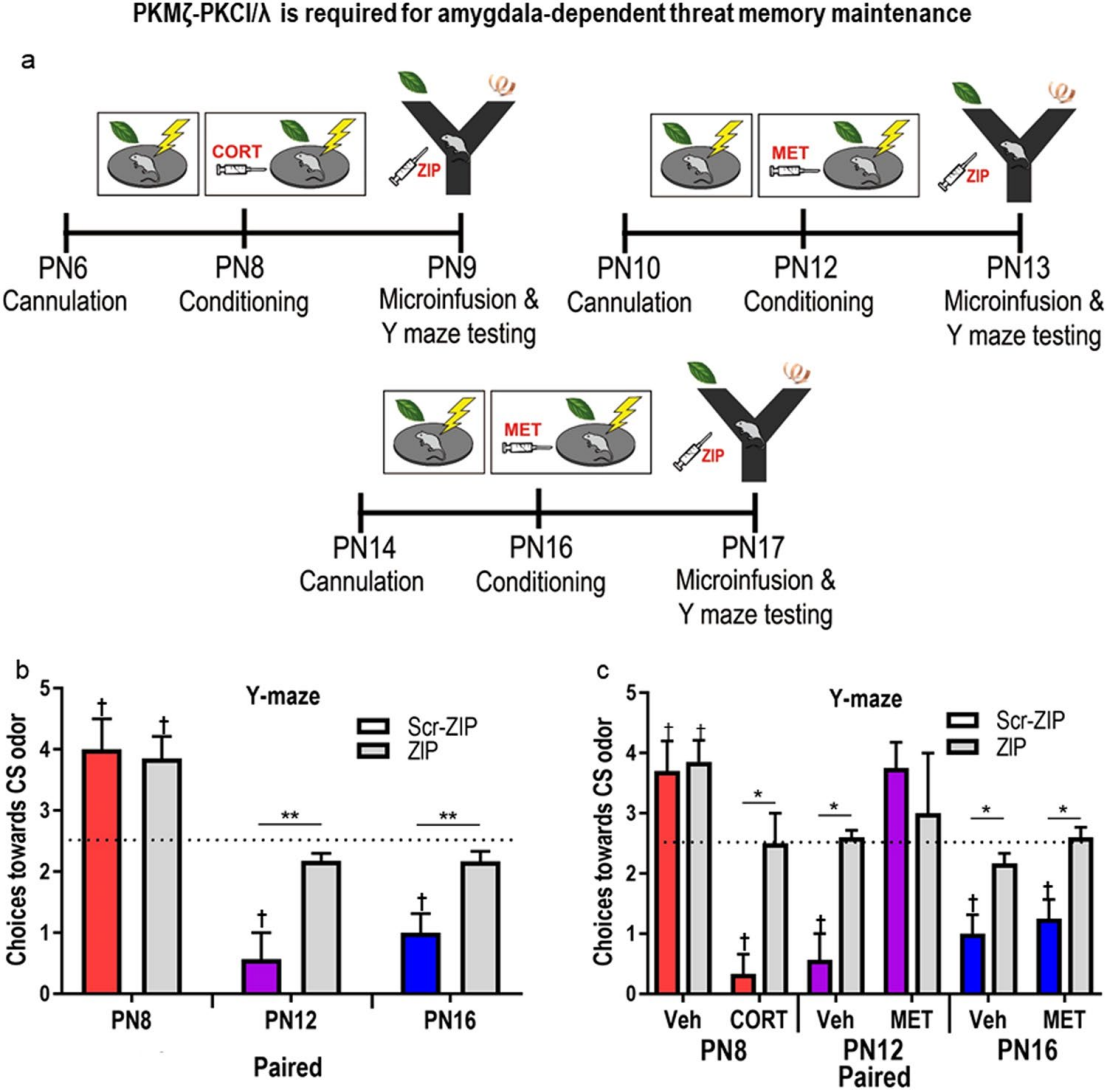
PKMζ-PKCι/λ is required for amygdala-dependent threat memory maintenance. To test causation, pups were cannulated bilaterally in the amygdala two days before conditioning (**a**) and administered ZIP or scrambled ZIP 2 hours before Y-maze testing. At baseline (no CORT manipulation), ZIP blocked amygdala-dependent threat memory expression in PN12 and PN16 pups that underwent odor-shock conditioning (**b**). ZIP administration blocked threat memory expression in pups that learned amygdala dependent threat following CORT manipulation (PN8 + CORT, PN12 + Veh, PN16 + MET, PN16 + Veh, (**c**). *P < 0.05, **p < 0.01, ***p < 0.001; † denotes significant difference from chance performance on Y-maze (dotted line), p < 0.05. Error bars indicate mean ± SEM. Veh, vehicle; MET, metyrapone; CORT, corticosterone. Representative histology confirming cannula placement is presented in Supplementary Figure 6.

Lastly, we determined the role of PKC-AMPAR trafficking in amygdala-dependent threat memory maintenance by administering bilateral microinfusions of ZIP (zeta inhibitory peptide; myristolated PKC Zeta, pseudosubstrate, ANASPEC, 1 mg/kg, 10 mM) or scrambled ZIP (Scr-ZIP, myristolated PKC Zeta, pseudosubstrate ZIP, Scrambled, ANASPEC, 1 mg/kg, 10 mM) into the basolateral amygdala 22 hours after conditioning (Fig. 4a, Supp. Fig. 6). ZIP has been used extensively as an inhibitor of PKMζ and PKCι/λ^52,55^ and reverses late-phase LTP^36,61,62^. Pups were tested on a Y-maze 2 hours after infusion, a time-frame shown to be critical for full PKMζ-PKCι/λ inhibition^35^. At baseline conditions (as in Experiment 1, no CORT manipulation), ZIP treatment prevented expression of learned avoidance in animals at PN12 and PN16, but not PN8 (Fig. 4b). A two-way ANOVA [drug (ZIP/Scr-ZIP) × age] revealed a significant interaction between the two factors (*F*_(2,25)_ = 6.6, *p* = 0.005) as well as main effects of age (*F*_(2,25)_ = 11.89, *p* = 0.0002) and drug (*F*_(2,25)_ = 9.985, *p* = 0.004). *Post hoc* tests confirmed the causal role of PKMζ-PKCι/λ activation in long-term memory maintenance when odor-shock produced an odor aversion: amygdala microinfusions of ZIP only impaired memory expression in pups that learned to avoid the odor (ZIP vs. Scr-ZIP: PN8, *p* = 0.97; PN12, *p* = 0.0098; PN16, *p* = 0.044). The effects of ZIP on memory expressed in the Y-maze choices differed between PN8 and PN12 and between PN8 and PN16, but not between PN12 and PN16 (ZIP: PN8 vs. PN12, *p* = 0.0021; PN8 vs. PN16, *p* = 0.0011; PN12 vs. PN16, *p* = 0.99).

PN8 pups administered ZIP chose the CS odor significantly above chance (*t*_(5)_ = 8, *p* = 0.0005), which was also observed when pups were administered scrambled ZIP (*t*_(6)_ = 6.874, p = 0.0005). In contrast, ZIP administration reversed the aversion towards the odor in PN12-conditioned pups, as these pups did not show a preference or aversion compared to chance (t(6) = 1.89, p = 0.10). However, scrambled ZIP administration failed to block the aversion memory in these pups, as they chose peppermint below chance (*t*_(6)_ = 5.203, *p* = 0.002). For pups conditioned at PN16, ZIP reversed the aversion behavior (*t*_(6)_ = 0.3536, *p* = 0.7358) while pups approached the odor significantly below chance following administration of scrambled ZIP (*t*_(6)_ = 5.809, *p* = 0.0021).

#### Blockade of PKC continues to be aligned with memory blockade, when the age of learning is switched on/off with CORT the previous day

As illustrated in Fig. 4c, we further tested the effects of ZIP blockade of memory by again dissociating memory molecules with changes in maturation by manipulating CORT to induce learning in PN8 pups, block learning in PN12 pups, and block CORT when it has no effect on learning in PN16 pups. Specifically, we tested the role of PKMζ-PKCι/λ in memory maintenance in conditions of CORT manipulation by administering ZIP or scrambled ZIP in the BLA (2 hrs before testing in Y-maze) of pups that received metyrapone (MET in graphs) (PN12/PN16), CORT (PN8 only) or vehicle (0.9% saline, i.p., all ages) prior to paired odor-shock conditioning the previous day. Overall, ZIP but not Scr-ZIP, disrupted expression of aversion memory at all ages, while pups that did not learn appeared unaffected by ZIP or Scr-ZIP. Three-way ANOVA between age, drug treatment at conditioning (vehicle vs. CORT manipulation) and drug treatment before testing (ZIP vs. Scr-ZIP) revealed an interaction between the three factors (*F*_(2,48)_ = 14.14, *p < *0.0001). Specifically, at PN16, ZIP prevented threat memory expression in all PN16 paired odor-shock groups, regardless of metyrapone presence during conditioning (PN16: paired + vehicle: ZIP vs. Scr-ZIP, *t*_(9)_ = 3.435, *p* = 0.007; PN16: paired + MET, ZIP vs. Scr-ZIP, *t*_(7)_ = 3.449, *p* = 0.011). CORT blockade by the CORT-inhibitor MET or other means does not alter learning in this age pup^58^. At PN12, ZIP disrupted memory expression in pups that learned, but did not alter expression in pups that had learning suppressed by metyrapone (PN12 paired + vehicle, ZIP vs. Scr-ZIP: *t*_(8)_ = 4.547, *p* = 0.001; PN12 paired + MET, ZIP vs. Scr-ZIP: *t*_(6)_ = 0.7071, *p* = 0.51) In PN8 pups, amygdala ZIP before testing disrupted memory expression in pups that received CORT before odor-shock paired conditioning, as these were the only PN8 pups that learned amygdala-dependent threat (PN8 paired + vehicle, ZIP vs. Scr-ZIP, *t*_(8)_ = 0.3959, *p* = 0.7096; paired + CORT, ZIP vs. Scr-ZIP, *t*_(8)_ = 3.622, *p* = 0.0151).

Our behavioral analysis shows that PN12 pups given metyrapone before conditioning and ZIP 22 hr later failed to show either preference or avoidance of the peppermint odor, as evidenced by choosing the odor at chance levels (*t*_(4)_ = 1.5, *p* = 0.208). However, PN12 pups administered MET and then Scr-ZIP retained a preference for the odor, choosing it significantly above chance at testing (*t*_(5)_ = 2.926, *p* = 0.0327). PN12 pups that learned (administered vehicle before conditioning) and that were given ZIP before testing the following day also failed to show avoidance of the odor (*t*_(6)_ = 1.162, *p* = 0.2894), while vehicle-injected pups given scrambled ZIP retained avoidance behavior of the CS, choosing it significantly below chance (*t*_(6)_ = 4.5, *p* = 0.0041).

In the oldest pups (PN16), metyrapone administration before training and ZIP before testing prevented expression of the learned threat avoidance behavior, (*t*_(5)_ = 0.796, *p* = 0.465). However, metyrapone-administered pups later given Scr-ZIP retain avoidance of the odor, choosing it significantly below chance (*t*_(4)_ = 6.5, *p* = 0.0029). Pups administered vehicle before conditioning fail to retain memory if administered ZIP before testing, choosing the odor at chance levels (*t*_(5)_ = 0.7906, *p* = 0.4650). However, pups that were conditioned and administered Scr-ZIP before testing retained threat avoidance memory, choosing the odor significantly below chance (*t*_(4)_ = 4.743, *p* = 0.009).

As in Fig. 3 and replicated here in Fig. 4, in PN8 pups that were too young to learn amygdala-dependent threat associations, amygdala ZIP administration failed to modulate avoidance memory in the Y-maze. Specifically, PN8 pups conditioned with vehicle chose the odor above chance at testing following both ZIP (*t*_(6)_ = 9.5, *p* < 0.0001) and Scr-ZIP (*t*_(7)_ = 7.937, *p* < 0.0001) administration. However, with precocious induction of learning in PN8 pups via CORT, this memory was retained if administered Scr-ZIP (*t*_(8)_ = 7.78, *p* < 0.0001); and this avoidance memory was blocked if pups were administered ZIP before testing (*t*_(7)_ = 0.851, *p* = 0.4229).

## Discussion

We build on the seminal infantile amnesia research by using odor-shock conditioning in developing infant rats and odor aversion testing the next day to assess the molecular memory mechanisms related to memory. We focus on the AMPAR and its GluA1/2 subunits known to support memory in adults, and present evidence of upstream control by PKMζ-PKCι/λ. We show consistent memory-associated increases in PKCι/λ at all ages, with the addition of increased PKMζ with the natural emergence of amygdala-dependent learning by PN12. At all ages, blockade of PKMζ-PKCι/λ with amygdala ZIP before testing blocks expression of what was learned the previous day. We also show consistent increases in GluA2 across development shown to be critical for synaptic plasticity and memory maintenance. However, only the oldest pups (PN16) showed memory-associated increases in GluA1 expression, which also increases in adults. In the young pup (PN12), the newly-acquired ability to learn and remember about threat was associated with GluA1 decrease. A similar profile of memory-associated GluA1 decrease was found in the youngest pups (PN8), which do not typically exhibit threat learning (Experiment 1) but can be precociously induced to learn via a CORT injection (Experiment 2). Taken together, these data suggest that threat memory during infancy is supported by novel molecular cascades involving at least two developmental transitions to the adult-like, memory-associated increases in GluA1, GluA2 and PKC isoforms^51,54^. In the first transition, we observed a developmental switch from GluA1 decrease to increase supporting memory maintenance as pups transition from sensitive period (<PN16) to post-sensitive period (>PN16). In the second transition, we observed a developmental switch in PKC isoform involvement in memory maintenance, with early memory (<PN10) associated with an increase in PKCι/λ and older pup memory (>PN10) associated with an increase in both PKMζ and PKCι/λ. Based on the existing literature, we suggest that as pups mature, the ability to remember threat cues evolves significantly during PN8-PN16, which can also aid in infant retention of memories in general.

This association between infant memory and the infant PKMζ-PKCι/λ→GluA1/2 signaling pathway was probed by capitalizing on a unique feature of infant learning, which is sensitive to amygdala levels of the stress hormone CORT. We show that memory molecules remain closely aligned with pup learning, even when learning was switched on/off by CORT manipulations in PN8 and PN12 pups (Fig. 2). Specifically, we pharmacologically blocked the shock-induced CORT increase that is naturally accomplished by the mother via social buffering in pups PN10–15^57^ and show that the PKMζ-PKCι/λ → GluA1/2 signaling pathway was not engaged when pups did not show threat memory expression. Additionally, the early induction of learning in very young pups (PN8) by CORT, noted above, highlights the induction of the underlying memory mechanisms engaged only when pups learned from odor-shock conditioning. Together, the results indicate that developmental shifts in memory encoding remain closely aligned with pup learning, even when learning was switched on/off by CORT manipulations in PN8 and PN12 pups. Importantly, MET injections to block CORT did not disrupt learning in the oldest pups (PN16)^58^ and memory-associated molecular changes were preserved 24 hr later. It should be noted that, although stress hormones have been shown to increase synaptic PKMζ and AMPA receptor trafficking^33,63^, our pup injections manipulating CORT occurred before acquisition and were timed to peak during acquisition and return to baseline soon post-conditioning^59^. While many reports show cytosolic PKMζ increases following synaptic plasticity and memory^54^ in adults, our PN8 CORT-treated pups did not show cytosolic changes.

Our results indicate major transformations of the AMPAR subunits in support of threat memory across early development. This AMPAR developmental transition is likely to have robust effects on memory development: AMPAR mediates most of the brain’s fast excitatory synaptic transmission to support rapid synaptic changes and is important for supporting neural plasticity associated with learning and memory but also forgetting^29,64^. Specifically, AMPAR are critical for determining synaptic strength through regulation/trafficking of the AMPAR subunits (GluA1–4) in and out of the membranes. Indeed, these trafficking modifications of the GluA subunits determine long term potentiation (LTP) and long term depression (LTD) believed to be critical for learning and memory^65^. The composition of the tetrameric AMPAR receptor’s four subunits overwhelmingly contain GluA2 subunits, most typically grouped as heteromers GluA1/2. Importantly, preventing synaptic removal of AMPAR containing GluA2 subunit prevents the natural forgetting of long-term memories^66–68^. Here we have shown a consistent increase in postsynaptic GluA2 in the amygdala when pups show avoidance memory across early development, including PN8 pups that receive CORT and PN12 and PN16 pups.

These data suggest that the subunit composition of the AMPA receptor is a critical feature of memory maintenance across development. As mentioned above, in all age pups, GluA2 increased in pups retaining a memory, similarly to adults^34,69^. However, there were changes in GluA1 in groups with memory that were not consistent with the adult literature. Specifically, the youngest pups showed a significant decrease in GluA1 associated with memory, including both with the precocious induction of threat learning by CORT in very young pups (PN8), and in older pups that have recently acquired the capacity for threat-learning (PN12). In the oldest pups (PN16), GluA1 expression becomes adult-like by exhibiting a memory-associated increase. The critical role of GluA1 in memory maintenance and LTP in adults suggests that younger pups’ memory maintenance has reduced reliance on GluA1. Considering that GluA1-containing AMPAR are essential for the expression of LTD^29,70^, and pups as young as PN8 show LTD^71^, it would appear that GluA1 is present and functional in these young pups that also show decreases in GluA1 with learning. Lower levels of GluA1 suggests poorer retention, as fear memories are impaired in GluA1-deficit mice^72^. These results potentially suggest that failure to upregulate GluA1 may underlie, in part, the rapid forgetting in early life, as increasing GluA1 subunits is known to be important for threat memory^72^.

The inability to see the characteristics of the adult-like increases in GluA1 in the younger pups is unlikely due to lack of available GluA1. First, our untrained control groups that did not learn showed significantly higher levels of GluA1 compared to groups that learned and expressed memory. Secondly, while the development of GluA1 and GluA2 have not been documented in the amygdala, it has been in the late-developing hippocampus^73^. Molecular development across brain areas can be potentially coordinated based on level of maturity and its functional development into a behavioral system. For example, fear conditioning supports amygdala-dependent cue learning and memory at PN10^18^, while hippocampus-dependent contextual learning emerges around PN17, although that learning is not retained for more than minutes until almost a week later^74–76^. There is a strong association between the development of hippocampal-dependent context memory and emergence of memory molecules: levels of GluA1 and GluA2 appear to have reached adult-like proportions by the time the threat-dependent memory system becomes active^77,78^. Hippocampal spatial navigation, which also emerges at about the same age, is associated with increases in AMPAR response duration, and transitions to more adult-like lower levels of GluA1 subunits and increases in GluA3 subunits. Indeed, this AMPAR subunit composition shift is also associated with increasing postsynaptic excitability and reduced threshold for activity-dependent synaptic potentiation^79^. The present results suggest a strong association between the development of cue learning and emergence of memory molecules.

The critical role of AMPARs in memory stabilization and maintenance is well established at both the systems level and within the LTP/LTD model^31^; during learning there is a rapid increase in the number of AMPAR subunits, as noted above. The upstream control of trafficking these receptors is more controversial, although it is becoming clearer that both PKMζ-PKCι/λ and protein kinase Ca2+/calmodulin-dependent protein kinase II (CaMKII) are important in memory maintenance in adults^37,38,56^. We focused on PKMζ-PKCι/λ because infant levels are high and remain relatively unchanged during development, while CaMKII levels are low^40,41,73^. A causal relationship between pup memory and PKMζ-PKCι/λ → GluA1/2 signaling cascade was demonstrated by perturbing PKMζ-PKCι/λ with an intra-amygdala ZIP infusion, which blocks memory and long term potentiation (LTP) in adults^55,80^. At all ages, pup memory was disrupted by the amygdala ZIP infusion before testing. Since ZIP targets both PKMζ-PKCι/λ and PKCι/λ is used when PKMζ is unavailable, it is difficult to know whether PKMζ or PKCι/λ was important, except in the youngest pups where precocious induction of threat learning by CORT was only associated with a significant PKCι/λ increase. This is the first study to assess infant amygdala PKMζ and PKCι/λ, and suggests sufficient levels of both isoforms are present in the developing amygdala, which is consistent with data derived from the developing hippocampus^73^. There is also evidence that these PKC isoforms, albeit present, might have a different developmental role^73,81^. Specifically, results based on the perirhinal cortex suggest the infant brain excitability might be more dependent on PKMζ and persistent activity of metabotropic glutamate receptor synaptic transmission^81^. Direct molecular control of forgetting may also be present in pups. Research using an odor preference conditioning paradigm that induces learning in all ages of rat pups, which is typically only retained for a day or two, demonstrated that memory could be retained longer when protein phosphatase calcineurin (CaN) was inhibited within the olfactory bulb 40 min after conditioning^82^. Future studies will be necessary in order to determine the relative contributions of the PKC isoforms and other mechanisms enhancing and attenuating memory across development.

## Conclusions/Clinical Implications

The seminal work on infantile amnesia indicated that this early life increased forgetting occurs in many learning domains, involves myriad brain areas and is represented across many species. As the exploration of neural mechanisms expands, it becomes clear that many developmental changes impact forgetting, including neurogenesis, functional connectivity between brain areas and environmental cues^10,11,15–17,38,39,47,83,84^. Here we focused on the development of molecular mechanisms supporting threat memory maintenance, which had previously been largely unknown. We document age-specific changes in the molecules supporting memory within the PKMζ-PKCι/λ → GluA1/2 signaling pathway, known to be involved in adult memory. Specifically, this molecular pathway transitions at least twice as pups mature and prepare for independent life. We suggest that these molecular memory transitions contribute to the memory deficits characteristic of infancy. Understanding age-specific mechanisms may help uncover the range of molecular and systems levels mechanisms impacting memory to help provide age- and mechanism-specific targets for intervention.

## Methods and Materials

### Experimental Design and Statistical Analysis

#### Objectives

The objective of this controlled laboratory experiment was to question whether adult-like molecular cascades supporting memory maintenance could be found in pups as they begin to learn and remember threat. We hypothesized that pups’ behavioral transition to adult-like learning about aversive stimuli at PN10 would be coincident with emergence of PKMζ-PKCι/λ and GluA1–2 involvement in amygdala-dependent learning, as has been shown in adults. We further hypothesized that CORT manipulations that switch threat learning on/off will produce a closely aligned switch in the corresponding changes in the molecular cascade associated with memory. This latter hypothesis was based on previous data showing a unique role for CORT, which is typically naturally modulated by the mother and controls pup learning to switch on/off, leading us to predict that molecular signaling pathways may be altered by these factors as well.

#### Research units

Measurements were taken of animal behavior and protein levels in the amygdala.

#### Randomization

Pups from each litter were counterbalanced via random assignment into conditioning groups (paired/unpaired/odor only) and learning modulation conditions (saline vehicle vs. pharmacological CORT inhibition/elevation), such that all data collection and processing was conducted randomly. To control for litter effects, a maximum of 1 male and 1 female were used from each litter per experimental condition.

#### Blinding

All memory retention testing and western blotting were conducted by experimenters blind to subjects’ conditioning group and acquisition interference condition via CORT.

#### Sample size

Based on previous studies with fear conditioning in adult rats, we estimated effect sizes expected to be obtained with odor-shock conditioning in rat pups, and subsequently conducted a power analysis to determine the minimum number of animals required per group to have sufficient statistical power to detect differences between our experimental groups.

#### Rules for stopping data collection

Power analysis was performed before the study was conducted to determine sample sizes; these were endpoints used for inclusion.

#### Data inclusion/exclusion criteria

Animals that failed to show acquisition of learning during conditioning were prospectively excluded from further behavior testing and biochemistry.

#### Outliers

Grubb’s test (α = 0.05) was used to determine statistical outliers.

#### Endpoints

Statistical tests were designed using the assumption of normal distribution and variance for control vs. treatment groups; this assumption was tested during statistical analyses.

#### Replicates

All behavior was done with lower n (~4–5/group) as pilot data and then replicated for formal study (including those with pharmacology manipulations).

#### Statistical Analysis

Statistical analysis was performed with GraphPad Prism v7.0. Group data were first tested for normality using the Shapiro-Wilks test. For all comparisons of protein levels and behavior without ZIP infusion (Experiments 1 & 2), we performed one-way or two-way ANOVA (age × condition) followed by Bonferroni-corrected *post hoc* pairwise comparisons. Experiments using ZIP were analyzed using three-way ANOVA (age × drug × condition) where appropriate and Student’s t-tests between ZIP-infused and scrambled ZIP-infused pups within each condition. All results are expressed as means ± SEM. P values < 0.05 were considered statistically significant; individual *p* values are indicated in main text of results.

### Subjects

Male and female Long-Evans rats born and bred at Nathan Kline Institute (originally from Harlan Laboratories) were used as subjects. They were housed in polypropylene cages (34 × 29 × 17 cm) with wood shavings, in a 20 ± 1 °C environment on a 12 h light/dark cycle. PN0, and litters were culled to 12 pups (6 males, 6 females) on PN1. Food and water were available *ad libitum*. All procedures (cannulation, conditioning, testing) occurred at PN6–9, PN10–13 or PN14-PN17. Only one male and one female were used from each litter per experimental group, and no animals were used more than one time. Pups were separated from their mother only for the duration of the conditioning sessions (maximum 1 hr). All procedures were approved by the Institutional Animal Care and Use Committee of Nathan Kline Institute and New York University, in accordance with guidelines from the National Institutes of Health.

### Pavlovian Odor-Shock Conditioning

For subjects PN13 and younger, conditioning was done in a custom-made conditioning chamber, which maintained pups at thermoneutral and delivered shock via an electrode attached to the base of the tail. Rats received 8 pairings of peppermint odor (McCormick Pure Peppermint, 30 s, delivered via flow dilution olfactometer, 2 L/min flow rate, 1:10 CS odor:air) co-terminating with an electric tail shock delivered through an electrode (1 s, 0.5 mA; Med Associates shock generator). Pups were placed individually in 600 mL clear plastic beakers for conditioning and given a 10 min acclimation period prior to the start of conditioning. Trials were separated by an inter-trial interval (ITI) of 4 min and all stimuli were administered via Ethovision (Noldus) software, which also recorded pup whole body activity levels to construct acquisition curves. Pups at this young age require conditioning via wire electrodes, rather than wire grid-chamber setup, because their small size would produce full body shocks using the chamber system. In addition, older pups cannot be conditioned using wire electrodes because they are more active and can interfere with the electrode placement. Despite these minor methodological differences, data from our laboratory has highlighted the robustness of threat learning circuitry across procedures^85–88^.

For subjects at PN16, rats were conditioned and tested in standard operant chambers (Coulbourn Instruments), housed within sound-attenuating chambers (Med Associates). Following a 10 min acclimation period, rats were conditioned with eight pairings of peppermint odor (McCormick Pure Peppermint, 30 s, delivered via flow dilution olfactometer, 2 L/min flow rate, 1:10 CS odor:air) co-terminating with a foot-shock (1 s, 0.6 mA). Foot-shock was administered via stainless steel grid floors of the conditioning chamber, which delivered electric shock (Coulbourn Instruments). Trials were separated by 4 min ITI, and all stimuli were administered via FreezeFrame (Actimetrics) software, which also automatically recorded freezing behavior to construct acquisition curves, although videos were observed by experimenters blind to experimental condition to verify behavior. Freezing was defined as the cessation of all movement except for that related to respiration and non-awake/rest body posture. Infant rats do not respond to a threat with freezing until after approximately the end of the second week of postnatal life, and prior to the emergence of freezing young pups respond to threat with whole body activation. Given the well-established learning principle that older infants encode information faster than younger infants^7^ and previous data on pup learning^58,89^, the stimulus strength and number of training pairs for each age were chosen based on the literature and pilot experiments that established equivalent levels of initial encoding. In unpaired control groups, the same procedure was carried out as described above, except the shock and odor were explicitly unpaired, with at least 60 s duration separating odor and shock onsets.

### Memory Retention Testing

Long-term memory assessment was conducted 24 hr after conditioning. Memory was assessed using 5 choice trials in a Y-maze with each arm containing either the CS odor (Kimwipe with 7.5 µL peppermint) or familiar clean bedding. The Y-maze consisted of an age-appropriately sized start box (PN9–13: 10 × 8.5 × 8 cm; PN17: 19 × 10 × 10 cm) and two arms (PN9–13: 24 × 8.5 × 8; PN17: 29 × 10 × 9.5 cm). Pups remained in the start box for 5 s before the door to each arm was opened and given 60 s to choose an arm. A response was considered a choice when the pup’s entire body entered the alleyway of the arm. The experimenter was blind to experimental condition.

### Drug Treatments

Pups were given an i.p. injection of either metyrapone (MET, CORT inhibitor; Sigma; 50 mg/kg), CORT (Sigma, 3 mg/kg) or vehicle (0.9% Saline). Dosage and timing of drug treatment for each age was based on the literature and pilot data^57,58^. Previous research on amygdala micro-injections has shown that these CORT effects on threat learning are dependent upon the amygdala^58,90^.

For PKC inhibition experiments, pups had bilateral amygdala cannuli implanted 2 days before conditioning (see Fig. 4) and cannula were lowered to coordinates previously identified for each age (PN6: AP, −0.8, ML: ±3.0, DV −5; PN10: AP, −0.8, ML: ±3.5, DV −5; PN14: AP, −0.8, ML: ±4.5, DV −6; all measurements relative to bregma). Following two days of recovery, 10 mM ZIP or Scrambled ZIP (in Tris-HCL, ANASPEC) was bilaterally infused into amygdalae (1 ul/min/side, rate) 2 hr before Y-maze testing. Cannula placement was confirmed with histology following testing.

### Amygdala Dissections

Rats were sacrificed by decapitation immediately after Y-maze testing. To assess protein expression, amygdalae were dissected using standard techniques, frozen on dry ice and stored at −80 °C until biochemistry preparation^91^.

### Tissue Preparation

#### Fractionation

Amygdalae were micro-dissected and prepared into cytosolic and synaptic fractions. Tissue was homogenized in a TEE (Tris 50 mM; EDTA 1 mM; EGTA 1 mM) buffer containing a SigmaFast, protease inhibitor cocktail (Sigma Aldrich). Tissues are homogenized in 200 µl of the TEE-homogenization buffer using 20 pumps with a motorized pestle. Homogenates are transferred to Eppendorf tubes and centrifuged at 3,000 g (5 min at 4o C), to remove unhomogenized tissue. The resulting supernatant is centrifuged at 100,000 g for 30 min. After ultracentrifugation, the supernatant is collected and stored as the cytosolic fraction. The remaining pellet is resuspended in 100 µl of homogenizing TEE buffer containing 0.001% Triton X-100, incubated on ice for 1 hr and then centrifuged at 100,000 g for 1 hr at 4 °C. The resulting pellet is resuspended in 50 µl of TEE buffer and stored as the synaptic fraction. The Pierce bicinchoninic acid assay (BCA) (Thermo Scientific, Rockford, IL) is used to determine protein concentration for each sample. Samples are reduced with 4x Laemmli sample buffer equivalent to 25% of the total volume of the sample and then boiled and stored frozen at −80 °C.

#### Western Blot

Samples (20 μg) are loaded onto a Tris/Gly 4–20% midi gel to resolve PKMζ (55 kDa, 1:1000), PKC (70 kDa, 1:1000) and GluA1–2 (100 kDa, 1:1000) (Cell Signaling Technology, MA, USA)^33^. Antibodies against α-tubulin (1:5000, Cell Signaling Technology, MA, USA) were used to estimate the total amount of proteins. Every gel contained 4 lanes loaded with the same control sample designated as all brain sample (ABS). ABS was used to standardize protein signals between gels. Gels are transferred to nitrocellulose membranes in IBlot® Dry Blotting System (Life Technologies; Carlsbad, CA) for 9 minutes. Nitrocellulose membranes are incubated in blocking solution containing 5% sucrose in Tris Buffered Saline with Tween-20 (TBST; 0.1% Tween-20 in TBS) for 30 min at room temperature. Membranes are incubated overnight in primary antibodies and then probed with Horseradish Peroxidase (HRP) conjugated secondary antibody. Membranes are incubated with Enhanced Chemiluminescence (ECL) substrate and exposed on CL-XPosure Film (Thermo Scientific; Rockford, IL). Films are scanned for quantification with NIH Image J. The amount of each protein was normalized for the amount of the corresponding α-tubulin detected in the sample. Group values were expressed as a percentage of a selected group average pixel density.

## Electronic supplementary material

Supplementary Information

## Data Availability

The data that support the findings of this study are available from the corresponding author upon request.
