# Supplementary material for: Developmental transitions in amygdala PKC isoforms and AMPA receptor expression associated with threat memory in infant rats
Source: Sci Rep. 2018 Oct 2;8:14679. doi: 10.1038/s41598-018-32762-y (PMC6168531; doi:10.1038/s41598-018-32762-y)
Supplement: Supplementary file 1 — Supplementary Information [file 41598_2018_32762_MOESM1_ESM.pdf]

Scientific Reports

Supplementary Information

Developmental transitions in amygdala PKC isoforms and AMPA receptor expression associated with threat memory in infant rats

Authors: Maya Opendak[1,2*], Roseanna M. Zanca[3,4], Eben Anane[2], Peter A. Serrano[3,4†], Regina M. Sullivan[1,2†]

Supplementary Figure 1.

GluA2 Blots (100 kDa)

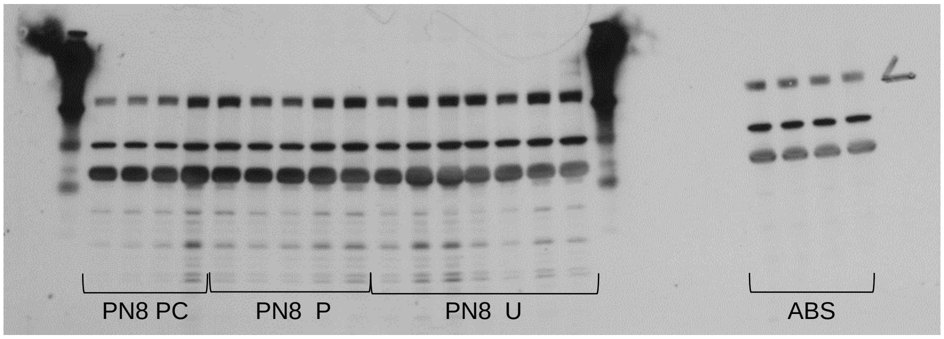

◄ GluA2

α-Tubulin comparison: Fig. 5c

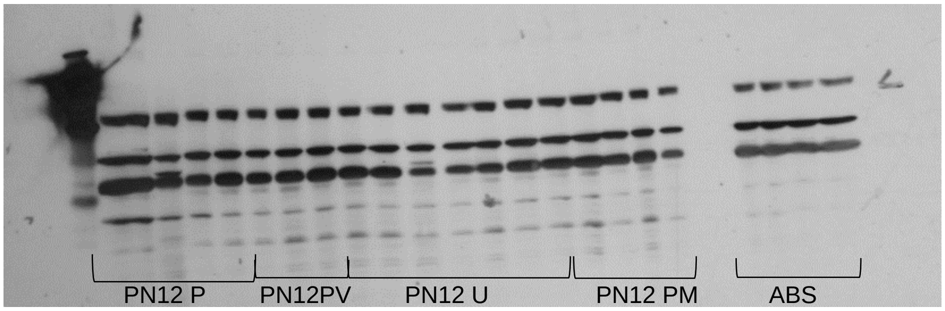

◄ GluA2

α-Tubulin comparison: Fig. 5d

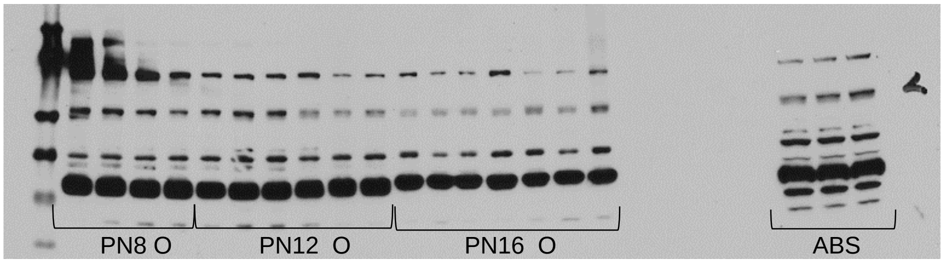

◄ GluA2

α-Tubulin comparison: Fig. 5f

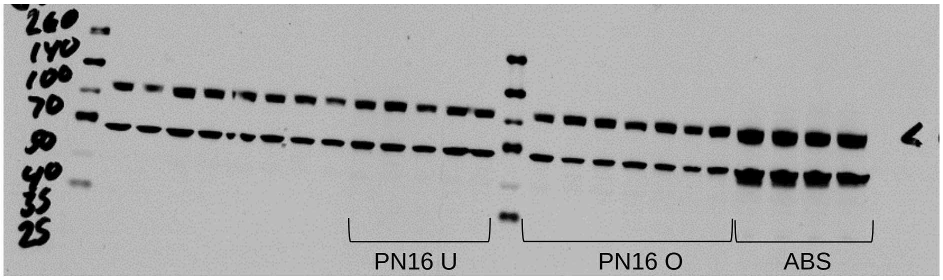

◄ GluA2

α-Tubulin comparison: Fig. 5k

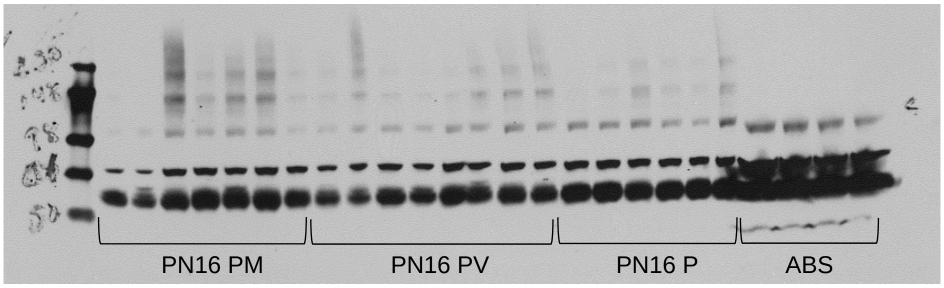

◄ GluA2

α-Tubulin comparison: Fig. 5l

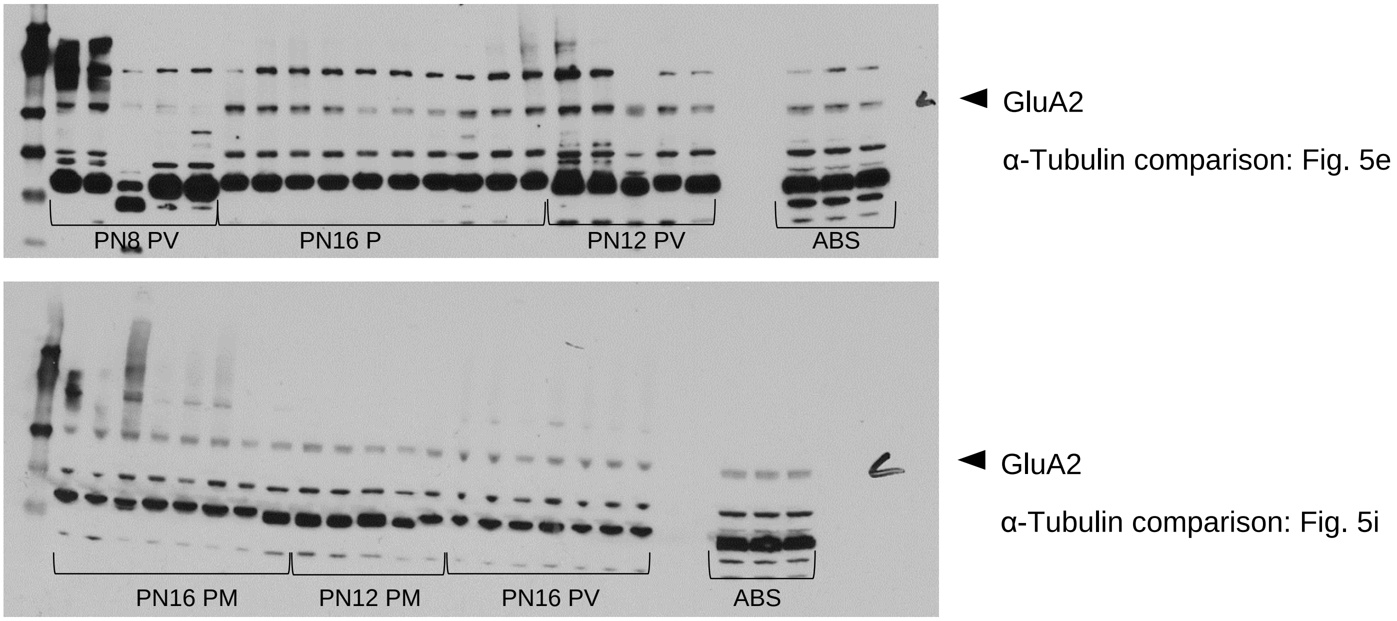

Supp. Figure 1. Western blot showing GluA2 (100 kDa, arrow) levels in amygdalae of pups conditioned at age postnatal day (PN)8, PN12 and PN16 and sac'd 24 hr later. P/U/O: Paired, Unpaired, Odor only conditioning. PC/PM/PV: Paired+CORT, Paired+Metyrapone, Paired+Vehicle. ABS: All brain sample, positive control. Empty lanes were loaded with 1x Laemmli Buffer. The same tubulin-corrected values were used for all markers probed. Comparisons were made across gels processed in parallel using samples derived from the same experiment. Unmarked blots were run as part of a separate study.

Supplementary Figure 2.

GluA1 Blots (100 kDa)

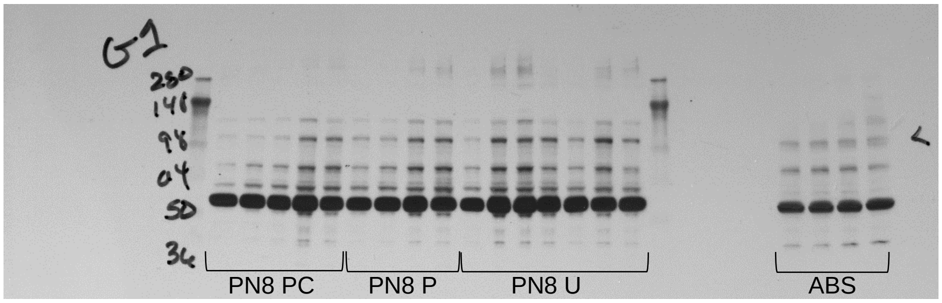

◄ GluA1

α-Tub comparison: Fig. 5c

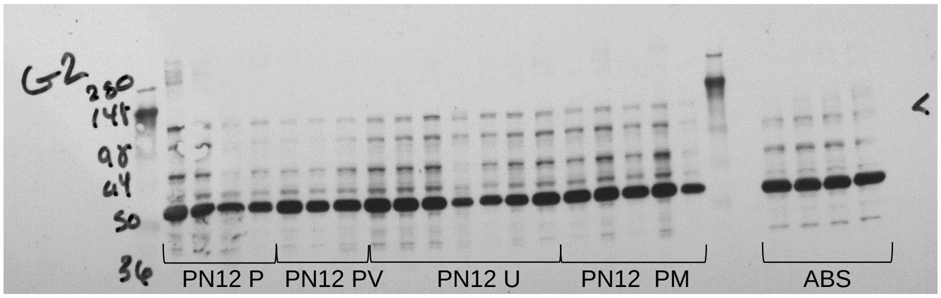

◄ GluA1

α-Tubulin comparison: Fig. 5d

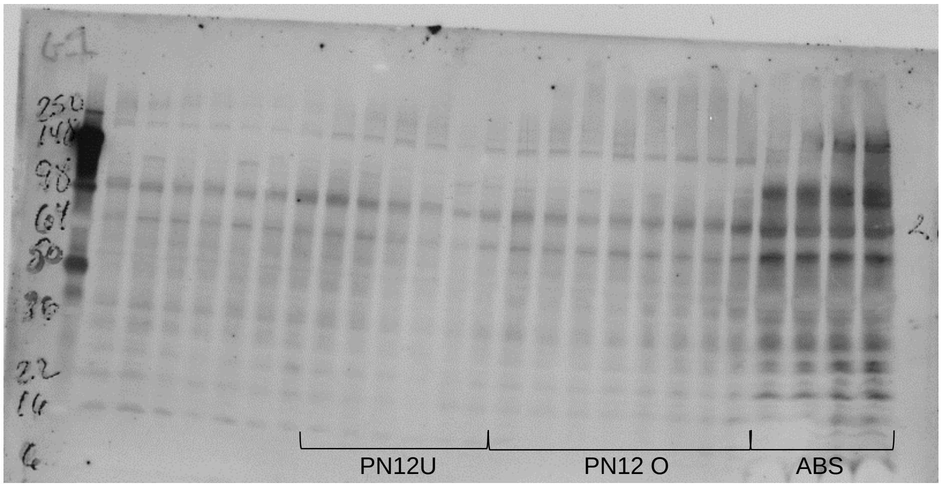

◄ GluA1

α-Tubulin comparison: Fig. 5j

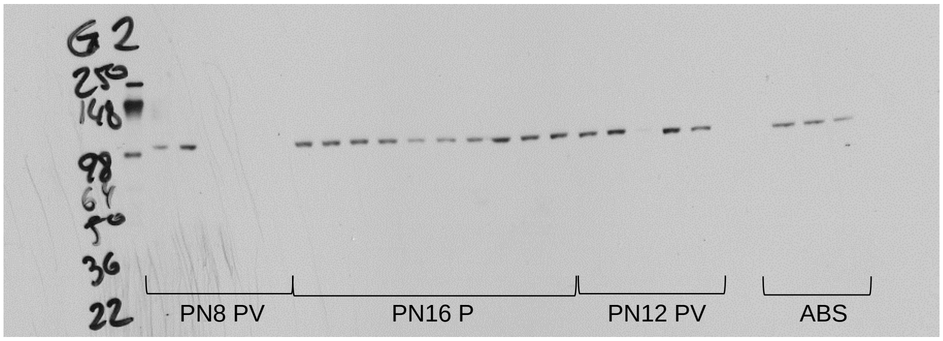

◄ GluA1

α-Tubulin comparison: Fig. 5e

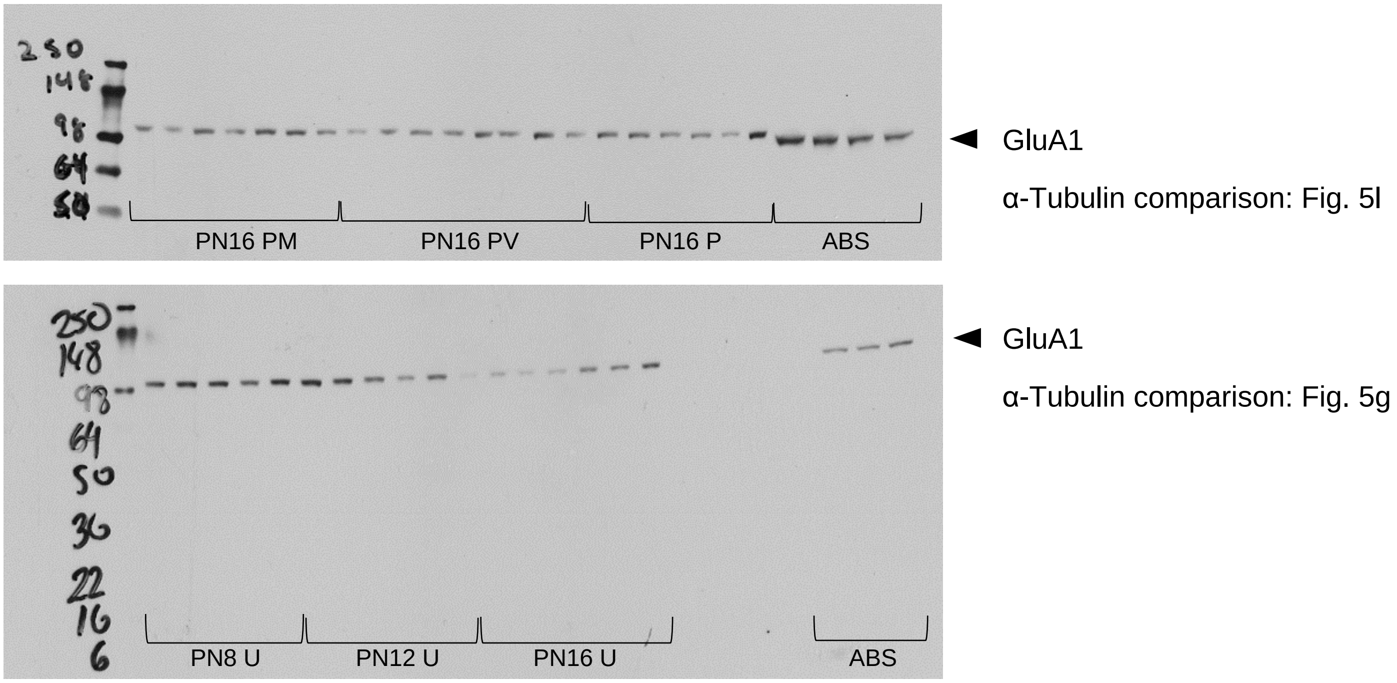

Supp. Figure 2. Western blot showing GluA1 (100 kDa, arrow) levels in amygdalae of pups conditioned at age postnatal day (PN)8, PN12 and PN16 and sac'd 24 hr later. P/U/O: Paired, Unpaired, Odor only conditioning. PC/PM/PV: Paired+CORT, Paired+Metyrapone, Paired+Vehicle. ABS: All brain sample, positive control. Empty lanes were loaded with 1x Laemmli Buffer. The same tubulin-corrected values were used for all markers probed. Comparisons were made across gels processed in parallel using samples derived from the same experiment. Unmarked blots were run as part of a separate study.

Supplementary Figure 3.

PKMζ Blots (55 kDa)

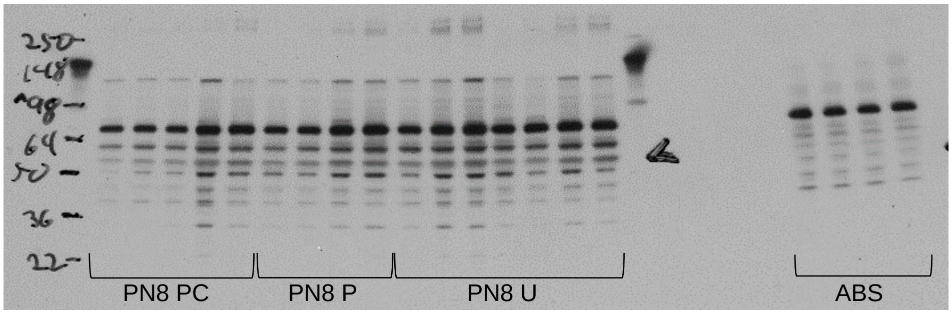

PKMζ

α-Tubulin comparison: Fig. 5c

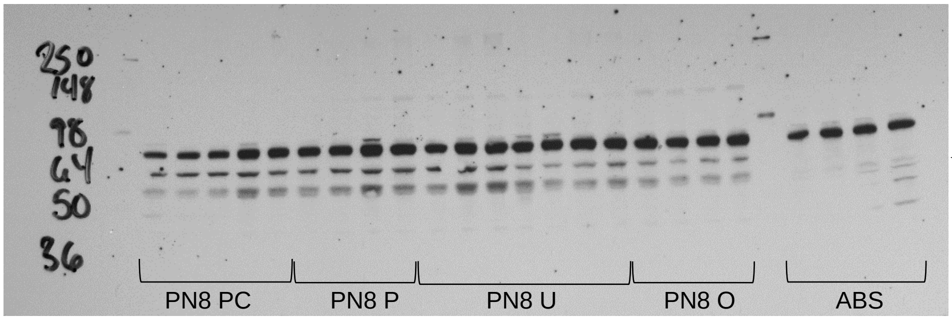

PKMζ

α-Tubulin comparison: Fig. 5b

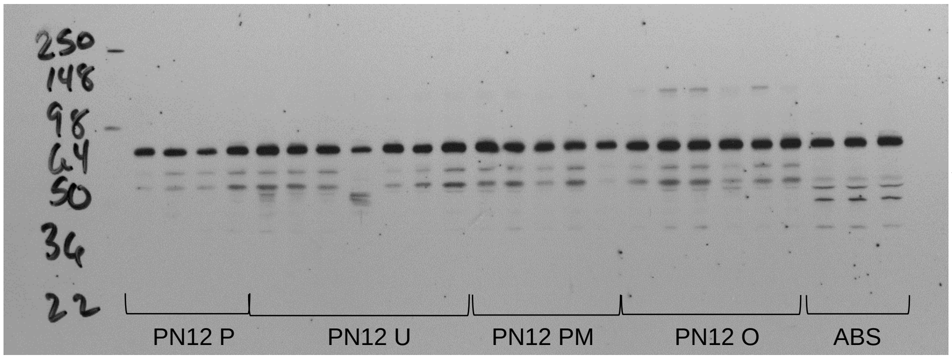

PKMζ

α-Tubulin comparison: Fig. 5a

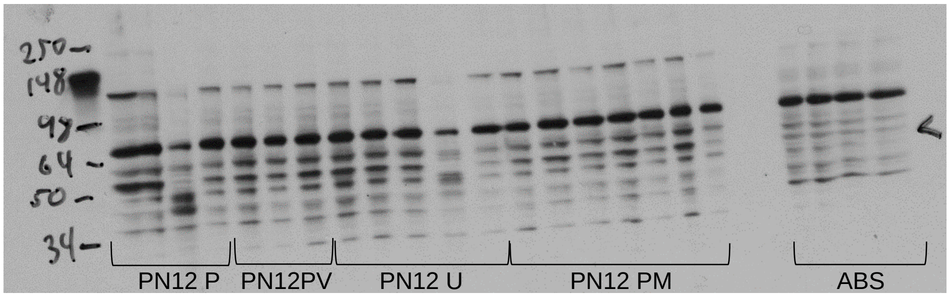

PKMζ

α-Tubulin comparison: Fig. 5d

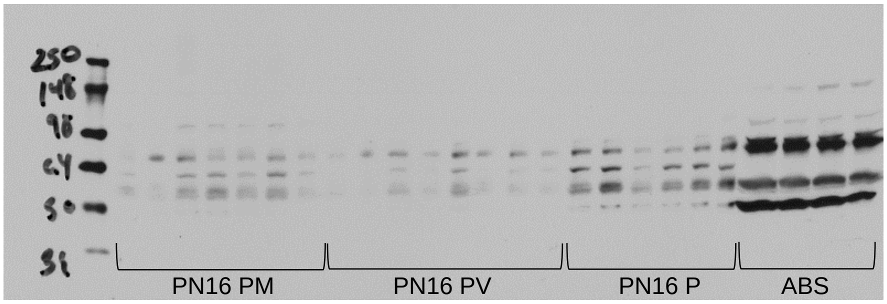

◄ PKMζ

α-Tubulin comparison: Fig. 5l

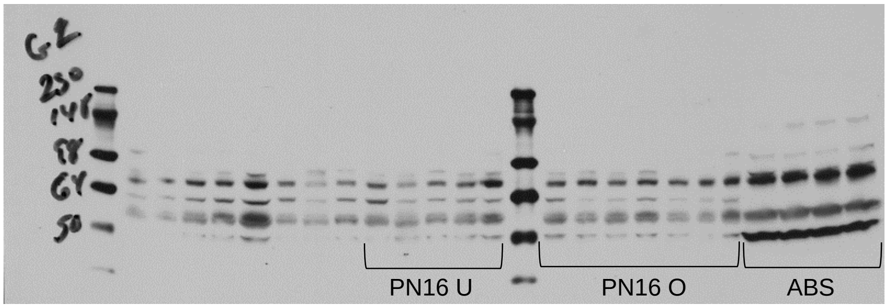

◄ PKMζ

α-Tubulin comparison: Fig. 5k

Supp. Figure 3. Western blot showing PKMζ (55 kDa, arrow) levels in amygdalae of pups conditioned at age postnatal day (PN)8, PN12 and PN16 and sac'd 24 hr later. P/U/O: Paired, Unpaired, Odor only conditioning. PC/PM/PV: Paired+CORT, Paired+Metyrapone, Paired+Vehicle. ABS: All brain sample, positive control. Empty lanes were loaded with 1x Laemmli Buffer. The same tubulin-corrected values were used for all markers probed. Comparisons were made across gels processed in parallel using samples derived from the same experiment. Unmarked blots were run as part of a separate study.

Supplementary Figure 4.

PKC ι/λ Blots (70 kDa)

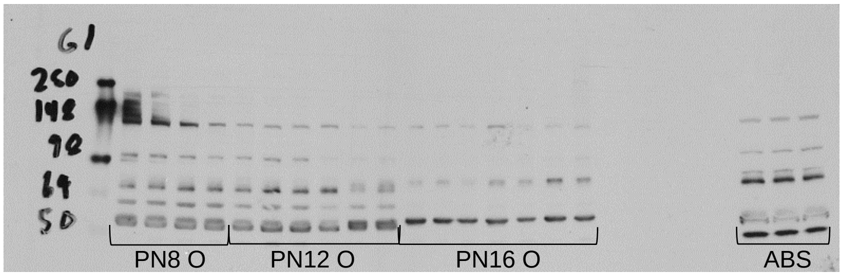

◄ PKC ι/λ

α-Tubulin comparison: Fig. 5f

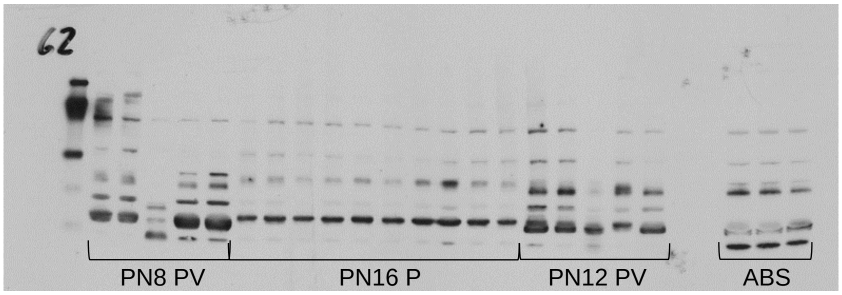

◄ PKC ι/λ

α-Tubulin comparison: Fig. 5e

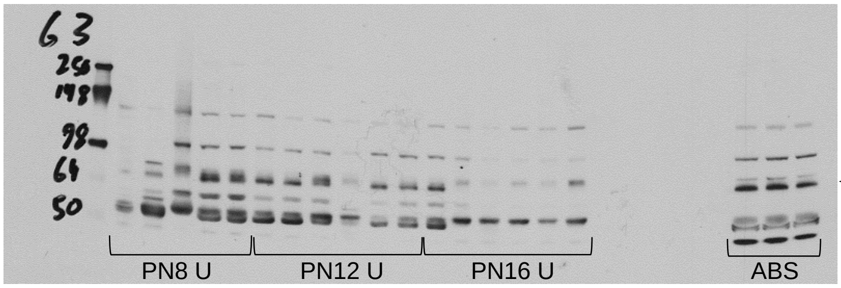

◄ PKC ι/λ

α-Tubulin comparison: Fig. 5g

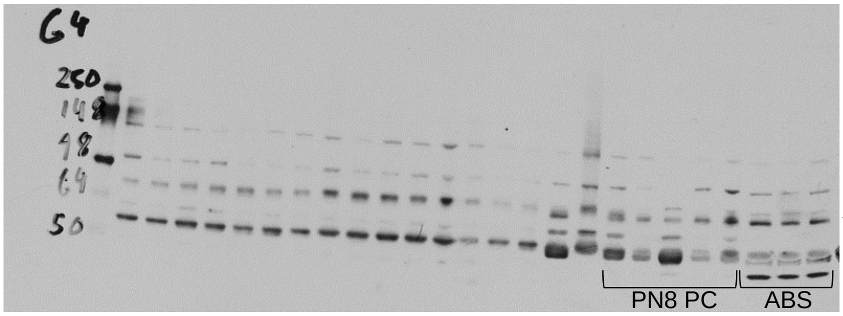

◄ PKC ι/λ

α-Tubulin comparison: Fig. 5h

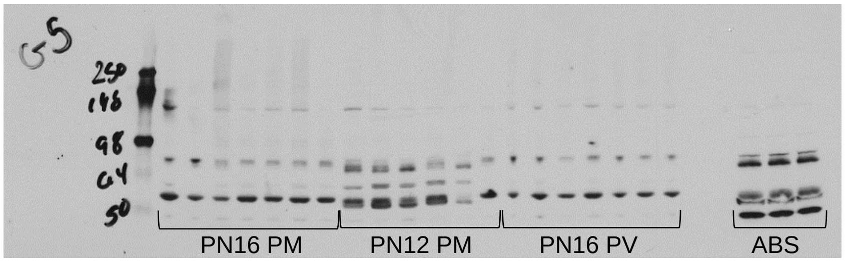

◄ PKC ι/λ

α-Tubulin comparison: Fig. 5i

Supp. Figure 4. Western blot showing PKCι/λ (70 kDa, arrow) levels in amygdalae of pups conditioned at age postnatal day (PN)8, PN12 and PN16 and sac'd 24 hr later. P/U/O: Paired, Unpaired, Odor only conditioning. PC/PM/PV: Paired+CORT, Paired+Metyrapone, Paired+Vehicle. ABS: All brain sample, positive control. Empty lanes were loaded with 1x Laemmli Buffer. The same tubulin-corrected values were used for all markers probed. Comparisons were made across gels processed in parallel using samples derived from the same experiment. Unmarked blots were run as part of a separate study.

Supplementary Figure 5.

α-Tubulin Blots (50 kDa)

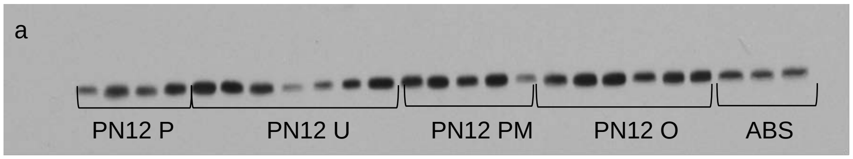

◄ α-Tubulin

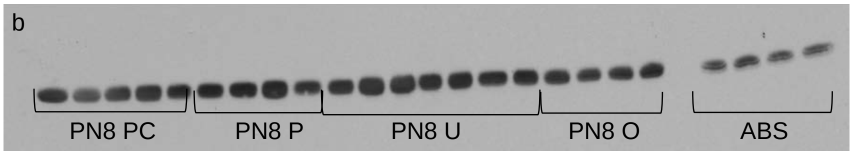

◄ α-Tubulin

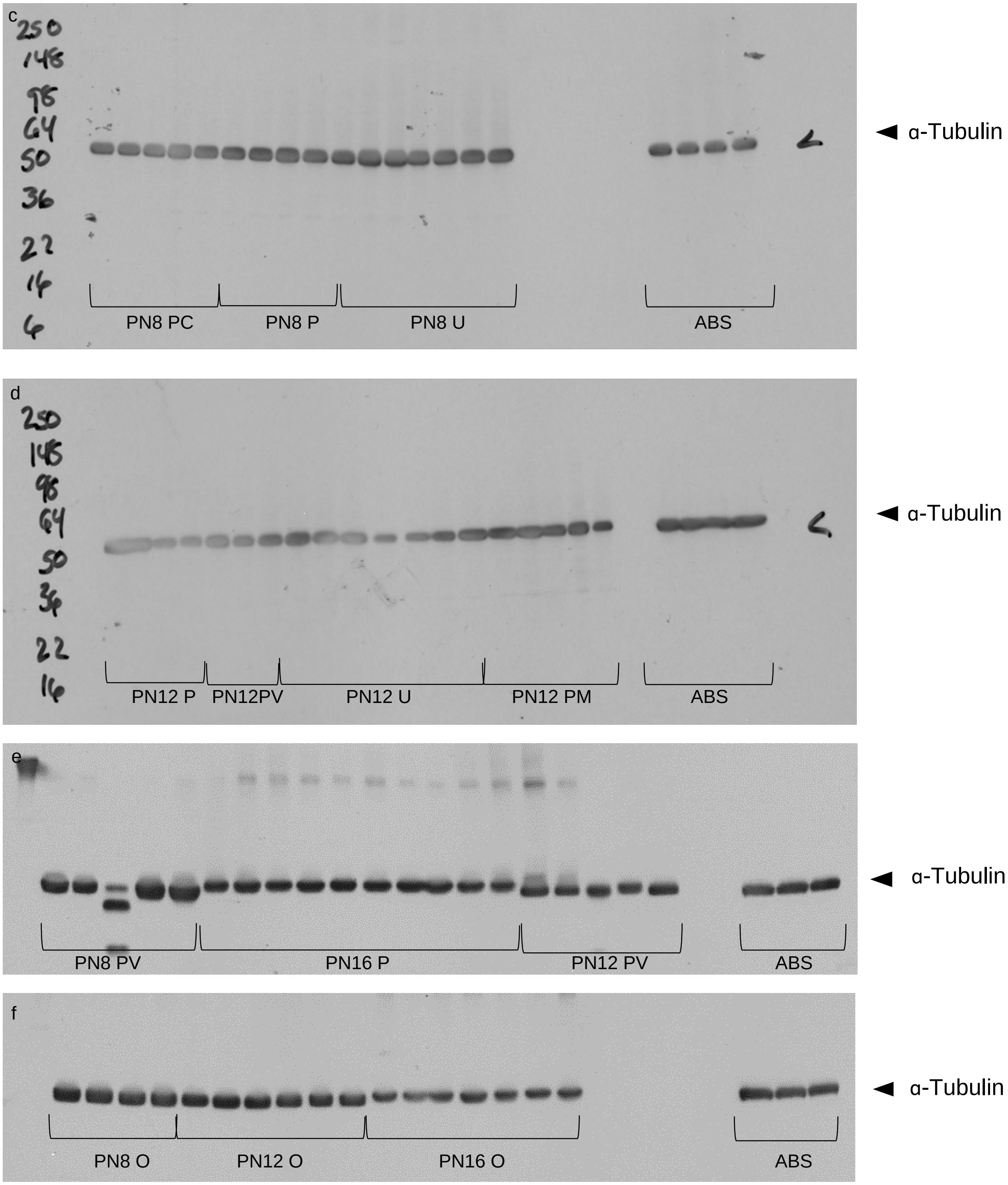
c
250
148
98
64
50
36
22
16
6
α-Tubulin
PN8 PC
PN8 P
PN8 U
ABS
d
250
148
98
64
50
36
22
16
α-Tubulin
PN12 P
PN12PV
PN12 U
PN12 PM
ABS
e
α-Tubulin
PN8 PV
PN16 P
PN12 PV
ABS
f
α-Tubulin
PN8 O
PN12 O
PN16 O
ABS

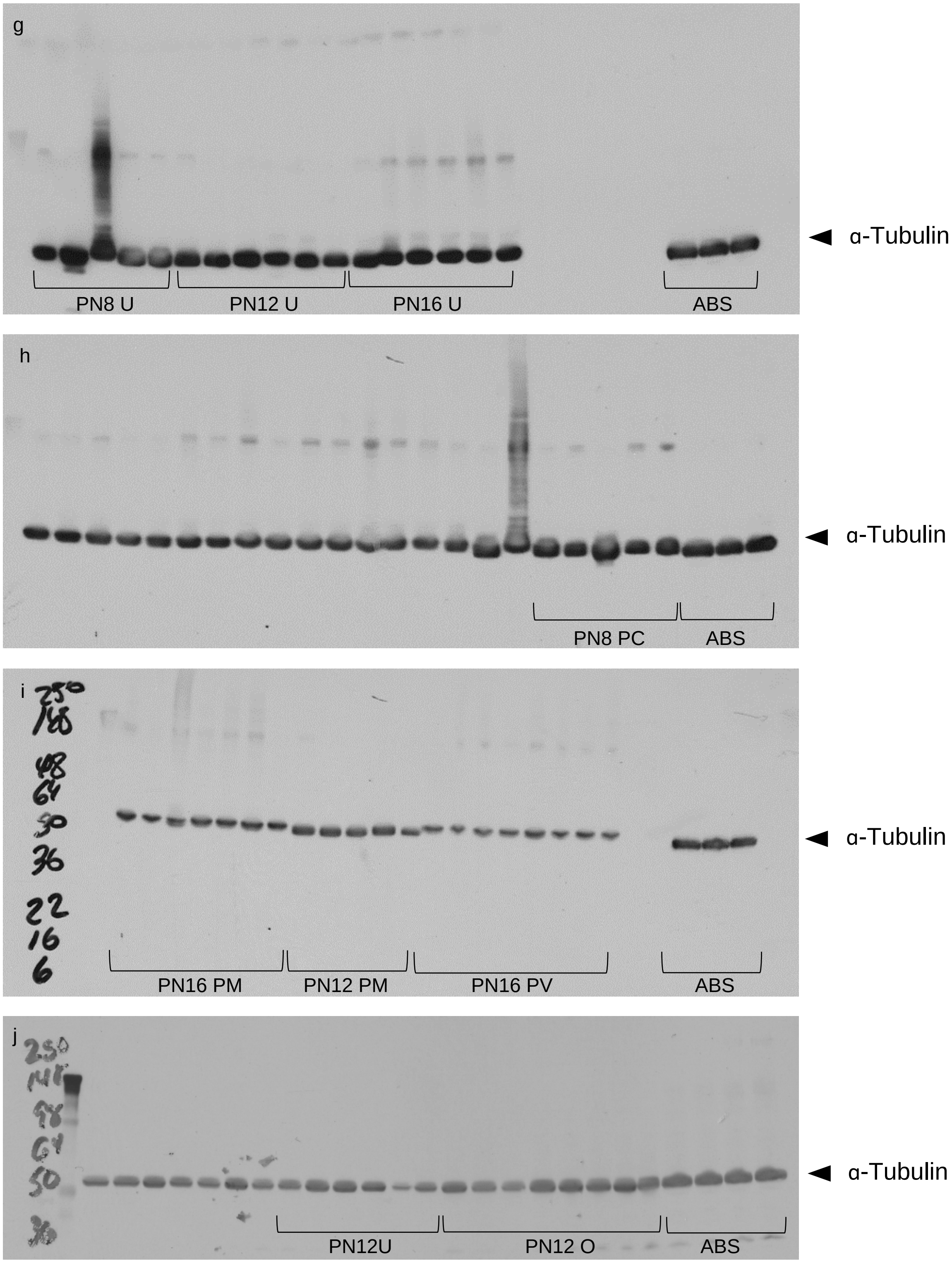
g
α-Tubulin
PN8 U
PN12 U
PN16 U
ABS
h
α-Tubulin
PN8 PC
ABS
i
α-Tubulin
PN16 PM
PN12 PM
PN16 PV
ABS
j
α-Tubulin
PN12U
PN12 O
ABS

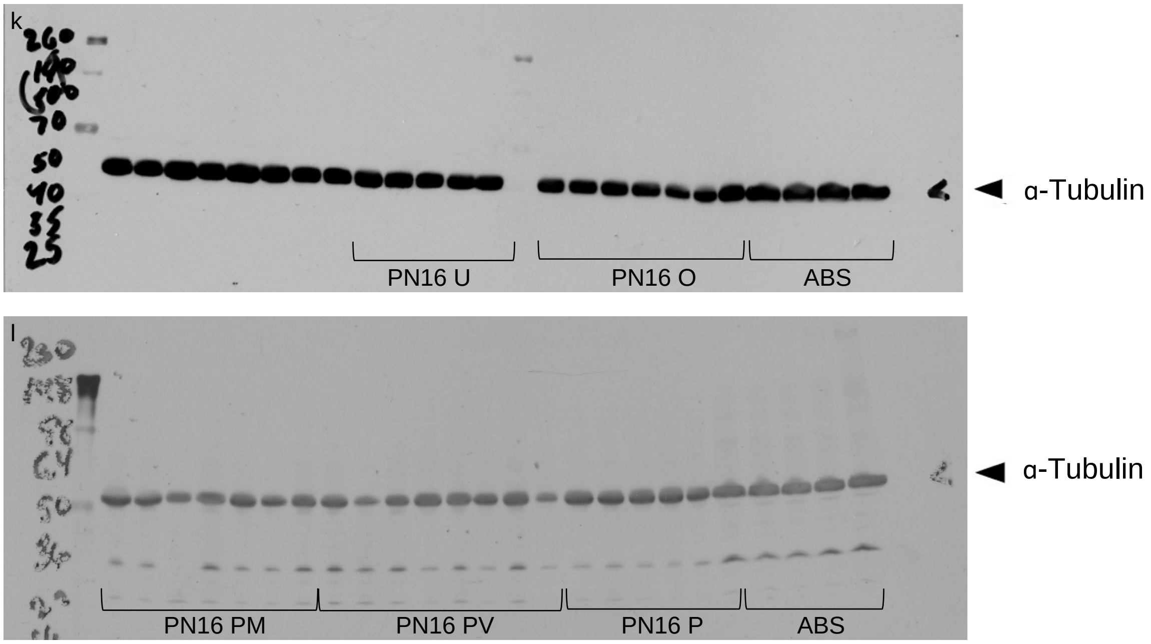

Supp. Figure 5. Western blot showing α-Tubulin (50 kDa, arrow) levels in amygdalae of pups conditioned at age postnatal day (PN)8, PN12 and PN16 and sac'd 24 hr later. P/U/O: Paired, Unpaired, Odor only conditioning. PC/PM/PV: Paired+CORT, Paired+Metyrapone, Paired+Vehicle. ABS: All brain sample, positive control. Empty lanes were loaded with 1x Laemmli Buffer. The same tubulin-corrected values were used for all markers probed. Comparisons were made across gels processed in parallel using samples derived from the same experiment. Unmarked blots were run as part of a separate study.

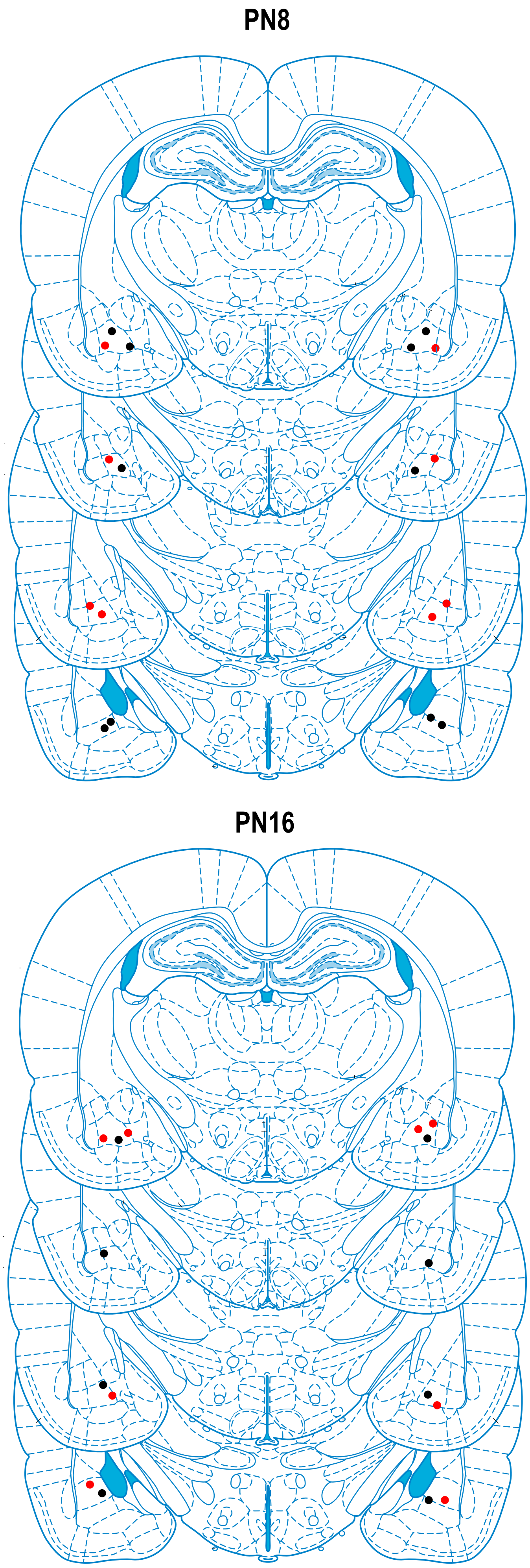

Supplementary Figure 6. Representative histology confirming cannula placement for pups receiving ZIP or Scrambled ZIP in Experiment 3 (Fig 4). Due to the large number of animals used, 4-5 representative pups per age per condition are shown here. Red dots, ZIP; black dots, Scr-ZIP.
